# Systematic Review of Diagnostic Approaches for Human Giardiasis: Unveiling Optimal Strategies

**DOI:** 10.3390/diagnostics14040364

**Published:** 2024-02-07

**Authors:** Bruno Vicente, Anna De Freitas, Marcus Freitas, Victor Midlej

**Affiliations:** 1 Laboratório de Biologia Estrutural, Instituto Oswaldo Cruz—Fiocruz, Rio de Janeiro 21040-900, Brazil; bruno-jpa1@hotmail.com (B.V.); annasilva@aluno.fiocruz.br (A.D.F.); marcuso2freitas@gmail.com (M.F.); 2 Programa de Pós-Graduação em Biologia Celular e Molecular, Instituto Oswaldo Cruz—Fiocruz, Rio de Janeiro 21040-900, Brazil; 3 Programa de Pós-Graduação em Biologia Parasitária, Instituto Oswaldo Cruz—Fiocruz, Rio de Janeiro 21040-900, Brazil

**Keywords:** *Giardia*, diagnostic, systematic review, PCR, microscopy, ELISA

## Abstract

Giardiasis, caused by the protozoan *Giardia intestinalis*, affects around 400 million people worldwide, emphasizing the critical need for accurate diagnosis to enhance human health, especially in children. Prolonged giardiasis in childhood can lead to intellectual deficits and other complications. A variety of diagnostic tools, including microscopic, immunological, and molecular methods, are available for detecting *G. intestinalis* infection. Choosing the most suitable method can be challenging due to the abundance of options. This systematic review assesses the reliability and applicability of these diagnostic modalities. Utilizing the Dimensions and Wordart platforms for data analysis, we focus on relevant literature addressing diagnostic methods for human giardiasis. Microscopic techniques, particularly Ritchie’s method, emerge as the primary choice, followed by enzyme-linked immunosorbent assay (ELISA) and polymerase chain reaction (PCR). PCR’s limited use is attributed to its high cost and infrastructure challenges in developing nations. In conclusion, our analysis supports microscopic methods as the gold standard for giardiasis diagnosis. However, in cases where symptoms persist despite a negative diagnosis, employing more sensitive diagnostic approaches is advisable.

## 1. Introduction

*Giardia intestinalis*, the causative agent of giardiasis, is a protozoan responsible for a prevalent infectious gastrointestinal disease affecting both children and adults on a global scale. This parasitic organism stands out as the most common cause of non-bacterial and non-viral intestinal diseases, contributing to an estimated 280 million cases annually [1]. First observed through a microscope in 1681 by Anthonie Van Leeuwenhok, it was formally described by VIlém Dušan Lambl in 1859 [2]. Recognizing the significant health and economic impact, particularly in developing nations, *G. duodenalis* was incorporated into The World Health Organization (WHO) Neglected Diseases Initiative in 2004 [3]. Giardiasis poses a heightened risk to preschool-aged children, daycare workers, travelers to developing nations, individuals with immunodeficiencies, and those who are malnourished [4]. Among the causes of intestinal diseases, *Giardia* is the protozoan most responsible for foodborne illnesses, making it a global concern due to its resulting morbidity and economic repercussions [5]. The situation is exacerbated in developing countries, where the prevalence of infected children can reach up to 60%, and those under 5 years old are particularly vulnerable to diarrhea-induced fatalities caused by giardiasis [6]. Infections during childhood by *G. intestinalis* significantly contribute to the emergence of an adult generation with deficits in both physical and cognitive development. This underscores the urgency of adopting a strategic approach to contain the spread of the disease [7,8]. Approximately 50% of cases remain asymptomatic, with re-infected children and adults being the primary agents responsible for propagating the infection within the population [2,9].

The detection and diagnosis of giardiasis present challenges for physicians, especially in regions distant from high-prevalence areas where a substantial number of asymptomatic patients further complicate prevalence determination. Symptoms, which closely mimic those of other parasitic diseases, can confound clinicians, potentially leading to the prescription of ineffective remedies [10]. The Foodborne Disease Active Surveillance Network (FoodNet) reported that out of 290 individuals infected with *Giardia*, about 25% did not receive appropriate therapeutic treatment. Inappropriate medications, such as fluoroquinolones (*n* = 23; 7.9%) and macrolides (*n* = 7; 2.4%), were administered in these cases [11]. Treatment for giardiasis primarily relies on nitroimidazole derivatives, notably Metronidazole, while microscopic detection of evolutionary forms (cysts and trophozoites) in feces remains the most common diagnostic method for parasite infection [12]. Additional techniques, including enzyme immunoassays, direct fluorescence, and real-time PCR, are employed for the detection of *Giardia* antigens or genetic material [13,14].

Several factors contribute to the complexity of *Giardia* diagnosis. The prevalence of asymptomatic individuals poses a challenge in determining the overall prevalence of the disease. For symptomatic patients, accurate detection hinges on the expertise of clinicians, the skills of technicians, and the availability of diagnostic resources, yet false-negative diagnoses may still occur [10]. In some countries, fecal samples are not routinely collected for individuals with diarrheal illnesses, further complicating diagnosis [15]. Certain nations, such as France, prioritize the search for bacterial or viral agents, with the parasitological examination of feces requiring additional time to be performed [16]. Currently, the lack of studies comparing different diagnostic methods, considering their respective advantages and disadvantages, hinders the dissemination of comprehensive knowledge. Consequently, there is a pressing need to conduct comparative analyses of diagnostic procedures to facilitate the actions of healthcare professionals, researchers, and organizations seeking to establish diagnostic laboratories. Such studies can guide professionals in selecting more efficient alternatives for giardiasis diagnosis, thereby enabling the administration of effective medications and ultimately safeguarding individual and collective health.

## 2. Materials and Methods

### 2.1. Protocol

This systematic review was conducted employing the “Preferred Reporting Items for a Systematic Review and Meta-Analysis” (PRISMA 2020 statement) as a touchstone in its completion [17]. The PRISMA checklist is accessible as Supplementary Materials (Table S3). This systematic review was not preregistered in any electronic database.

The same authors independently carried out the comprehensive literature search, the election of the papers, and the assessment of their quality. Any discrepancy between the reviewers was solved through a consensus meeting.

### 2.2. Repository

The keyword research was conducted on the online platform https://www.dimensions.ai/. Dimensions is a comprehensive web-based tool equipped with diverse functionalities for the retrieval of scientific papers, clinical cases, and more. Boasting a repository of 140 million publications, the platform offers a free version, ensuring accessibility for a broad audience.

### 2.3. Data Collection and Report Selection

**Figure 1:**
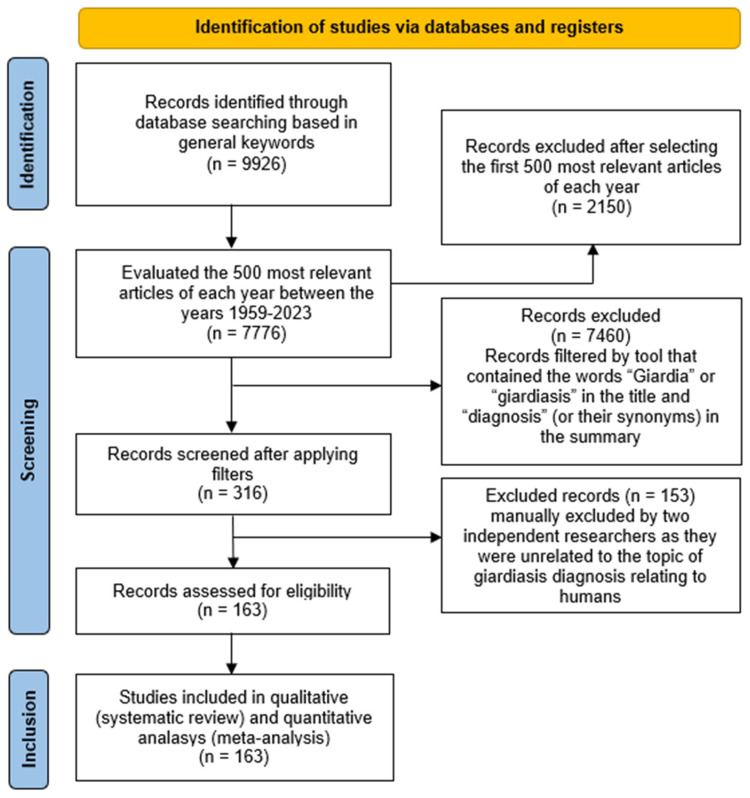
Comprehensive overview of the study selection process for the systematic review.

The analysis involved the collection of data on microscopic, immunological, and biomolecular diagnostic approaches. The methodology employed in this study centered on the identification of English keywords. The chosen keywords were as follows: “(diagnosis OR diagnostic) AND (giardiasis OR *Giardia intestinalis* OR *Giardia lamblia* OR *Giardia duodenalis*) AND (human) AND (method OR methodologies).” However, the initial use of overly broad keywords resulted in an extensive number of publications (9926 articles). It is presumed that this abundance encompassed topics deviating from the core theme of the article. Consequently, two rounds of evaluations were undertaken. The first phase involved the selection of the 500 most pertinent articles for each year spanning from 1959 to 2023. These articles were filtered based on the inclusion of the terms “*Giardia*” or “giardiasis” in the title and “diagnosis” or its synonyms in the abstract. The subsequent evaluation included a detailed examination of the abstracts of the filtered articles (316), with the exclusion of those unrelated to the diagnostic theme in human health (Figure 1). The data were categorized into three primary groups: Microscopy, Immune, and Molecular.

### 2.4. Word Cloud

The platform www.wordart.com was utilized to generate a visual representation of the most frequently occurring words in the abstracts of the selected articles. This tool automatically eliminates numbers and other linguistic connectives to highlight the core terms.

### 2.5. Data Compilation

Data were collected based on specific parameters including specificity, sensitivity, country of origin, year of publication, methods employed, type of sample, staining techniques, and types of antigens/kits. The amassed data were compiled and presented through graphical representations generated with the assistance of GraphPad Prism 8.4.3 (686) software.

## 3. Results

**Figure 2:**
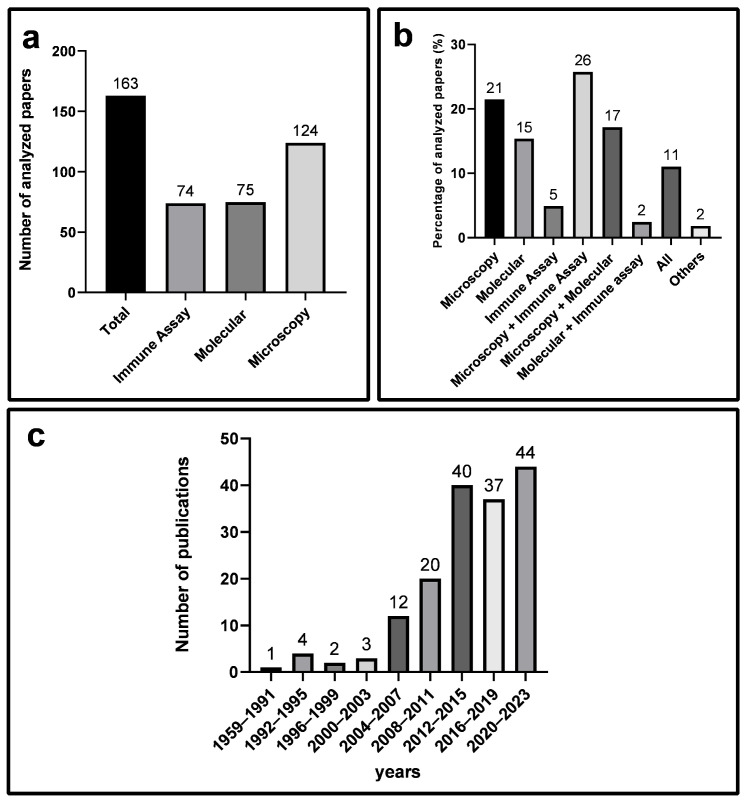
Comprehensive analysis of selected articles on diagnostic methods for Giardiasis. (**a**) Presents the number of articles selected for this study, categorized by method groups: Immune Assay, Molecular, and Microscopy. (**b**) Highlights the percentage of each methodology’s usage in the analyzed articles, considering whether it is employed independently or in combination with other techniques. (**c**) Illustrates the number of articles published every 4 years over a 64-year period.

A total of 163 articles addressing diagnostic methods for giardiasis were subjected to analysis (Figure 1; Supplementary Table S1). These selected articles were categorized into three groups based on diagnostic methodologies: microscopy, immune methods, and molecular methods (Figure 2a). However, recognizing that certain articles addressed multiple methodologies, we opted to quantify the percentage of isolated and combined usage of these approaches. Microscopy emerged as the most frequently used isolated methodology, constituting 21% of the publications (Figure 2b). Moreover, microscopic techniques were found in conjunction with 26% and 17% of publications addressing immune and molecular methods, respectively. An examination of articles published between 1984 and 2023 revealed a progressive increase in their numbers over the years (Figure 2c).

Word clouds, serving as a potential tool for the analysis of event or term frequencies within a set of articles, were generated for all selected abstracts using the wordart.com platform (Supplementary Figure S1a–c). This analysis maintained the three diagnostic method groups: Microscopy (Figure S1a), Immune (Figure S1b), and Molecular (Figure S1c). Notably, the Molecular group exhibited prevalent terms such as “sample” and “PCR” (polymerase chain reaction) underscoring their significance in articles related to the theme. Conversely, both the Microscopy and Immune groups featured words associated with all three techniques, indicating that articles explore not only the primary technique but also others for result comparison.

**Figure 3:**
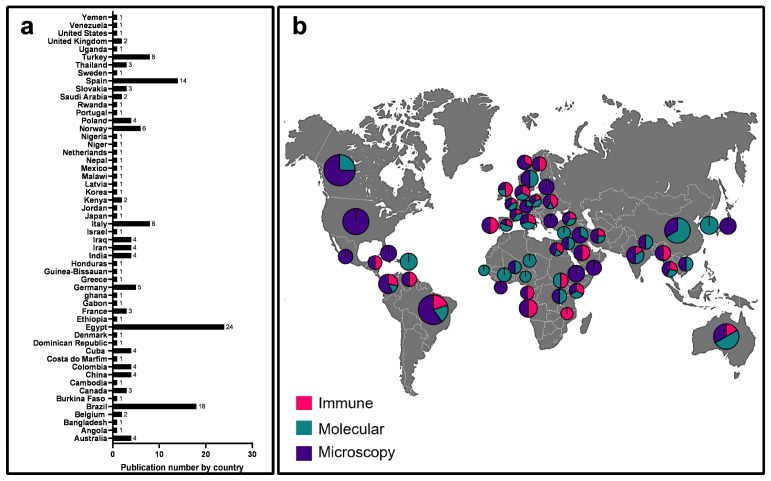
Analysis of the number of articles published in each country. (**a**) Articles were categorized according to the country of publication. (**b**) Proportion of methodologies used in each country.

The quantification of publications by country and the types of diagnostic techniques employed in each country was meticulously carried out (Figure 3; Supplementary Table S2). For the representation of each publication and its respective country, the data were scrutinized by analyzing the publication to determine where the samples were collected. This information was obtained either from the title or the methods section of each publication. In cases where this information was not available, we represented all different countries of the authors. Egypt led in the number of published literature on giardiasis diagnosis with 24 publications, followed by Brazil with 18, and Spain with 14 (Figure 3a). The distribution of methodologies and the proportion of each methodology by country are clearly discernible (Figure 3b).

**Figure 4:**
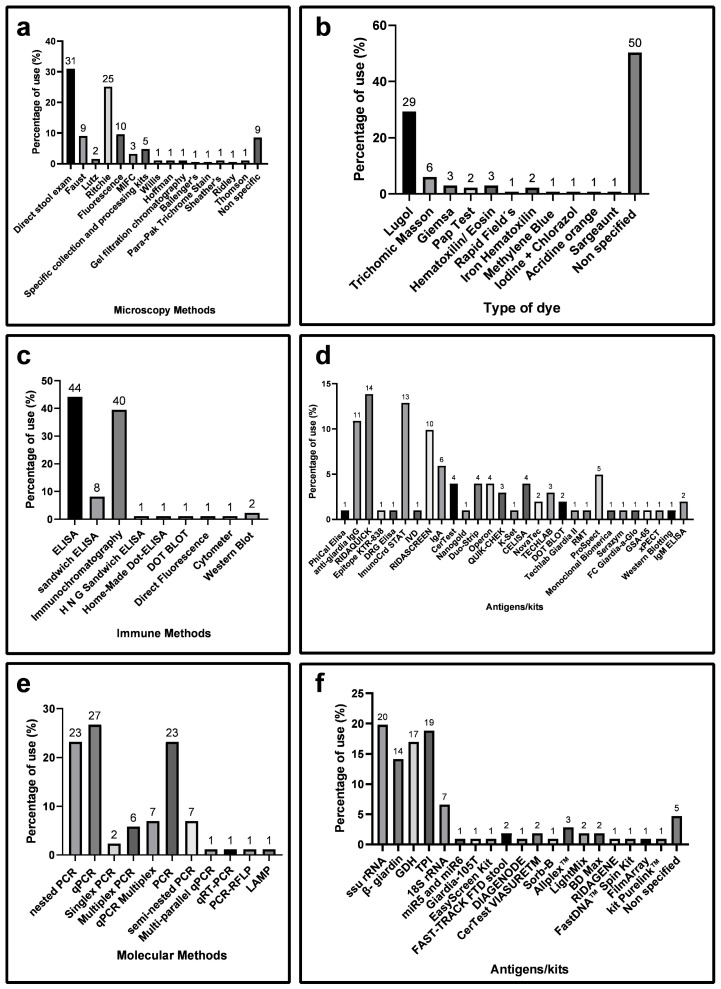
Detailed analysis of the specific diagnostic method for giardiasis from selected articles. (**a**) Illustrates the variety of microscopic diagnostic methods employed in giardiasis diagnosis. Articles that did not specify the methods used or recommended were categorized as “unspecified.” (**b**) Provides details on the types of dyes applied to samples processed by microscopic methods. (**c**) Showcases the most prevalent immunoassays in giardiasis diagnosis. (**d**) Identifies the types of antigens and kits used in the analyzed articles related to immunological assays. (**e**) Highlights the most frequently used molecular assays in giardiasis diagnosis. (**f**) Addresses the genetic targets employed in giardiasis diagnosis by molecular methods. MIFC: Merthiolate-Iodine Formaldehyde Concentration; RMT: rapid membrane test; LAMP: loop-mediated isothermal amplification; TPI: triose-phosphate isomerase; GDH: glutamate dehydrogenase; SSU rRNA: small subunit ribosomal RNA.

The three groups of diagnostic methods were independently analyzed. In the Microscopy group, direct observation emerged as the most utilized technique at 32%, followed by the Ritchie method (concentration method: formaldehyde-ether) at 25% (Figure 4a). Given that biological structures are nearly colorless under the microscope, the use of dyes becomes essential. Various dyes were employed to aid professionals in identifying cysts amidst fecal debris [12]. In Figure 4b, Lugol’s iodine was identified as the most used dye, appearing in 29% of the articles; however, 50% of the articles did not specify whether they employed any dye or analyzed the samples directly.

**Figure 5:**
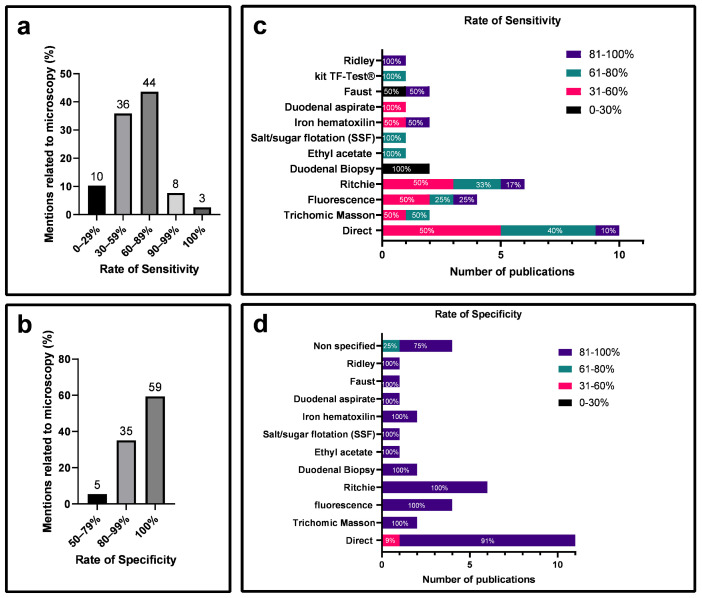
Sensitivity and specificity rates analysis for the microscopic diagnostic method of giardiasis based on selected articles. (**a**) Categorization of studies according to sensitivity rates. (**b**) Categorization of studies according to specificity rates. (**c**) Percentage representation of sensitivity rates for techniques employed in the microscopic method. (**d**) Percentage representation of specificity rates for techniques utilized in the microscopic method.

Sensitivity and diagnostic specificity are pivotal parameters when considering the choice of a method. Therefore, the specificity and sensitivity of methods related to giardiasis diagnosis were evaluated (Figure 5). Among the selected articles in the Microscopy group, 44% exhibited sensitivity between 60 and 89% (Figure 5a), and 59% showed 100% specificity (Figure 5b). Direct examination had 50% of the articles with sensitivity above 61% (Figure 5c) and 91% with a specificity above 81% (Figure 5d). The Ritchie technique emerged as the second-highest performing diagnostic method, with 50% of articles showing sensitivity above 61% (Figure 5c) and 100% with a specificity above 81% (Figure 5d).

In the Immune group, the enzyme-linked immunosorbent assay (ELISA) technique was the most used, followed by the immunochromatographic technique (Figure 4c). Since immune assays can target various antigens, we examined the target antigens in the selected studies, with RIDAQUICK, ImunoCard STAT!, and RIDASCREEN kits being predominantly utilized for giardiasis diagnosis. The most employed detection was anti-giardia IgG (Figure 4d).

**Figure 6:**
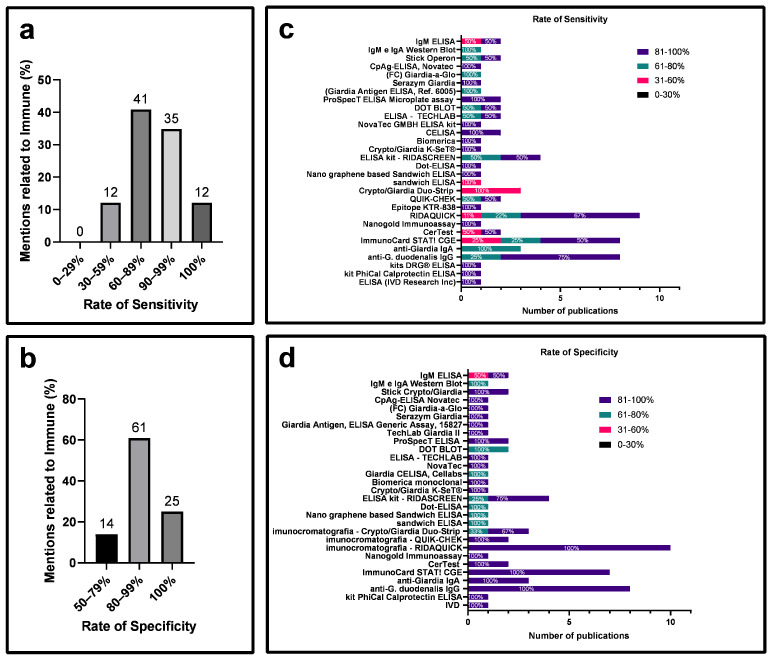
Analysis of specificity and sensitivity rates for the immunoassay diagnostic method of giardiasis from selected articles. (**a**) Categorization of studies on giardiasis immunoassay diagnostics based on sensitivity rates. (**b**) Categorization of studies on giardiasis immunoassay diagnostics based on specificity rates. (**c**) Percentage representation of sensitivity rates for techniques employed in the immunoassay method. (**d**) Percentage representation of specificity rates for techniques utilized in the immunoassay method.

Regarding sensitivity, 41% of the articles reported rates between 60 and 89%, and 12% reported rates between 90 and 99% (Figure 6a). In terms of specificity rates, 60% of the articles had rates between 80 and 99%, and 25% had rates of 100%. A comparison of the sensitivity and specificity of each kit/antigen used revealed that the RIDAQUICK immunochromatographic kit exhibited 48% of articles with sensitivity between 81 and 100%, and 100% with a specificity between 81 to 100% (Figure 6c,d).

PCR was the final diagnostic method scrutinized in this review. Figure 3b and Figure 4e present the collected data related to this molecular tool as a diagnostic method. Notably, the qPCR technique was the most employed with 23 reports, followed by the nested PCR and PCR techniques, each with 20 reports (Figure 4e). The genetic targets most commonly used for giardiasis diagnosis were summarized, with the small subunit ribosomal ribonucleic acid (SSU rRNA) gene being the predominant choice, followed by Triose-phosphate isomerase (TPI) and Glutamate dehydrogenase (GDH) (Figure 4f).

**Figure 7:**
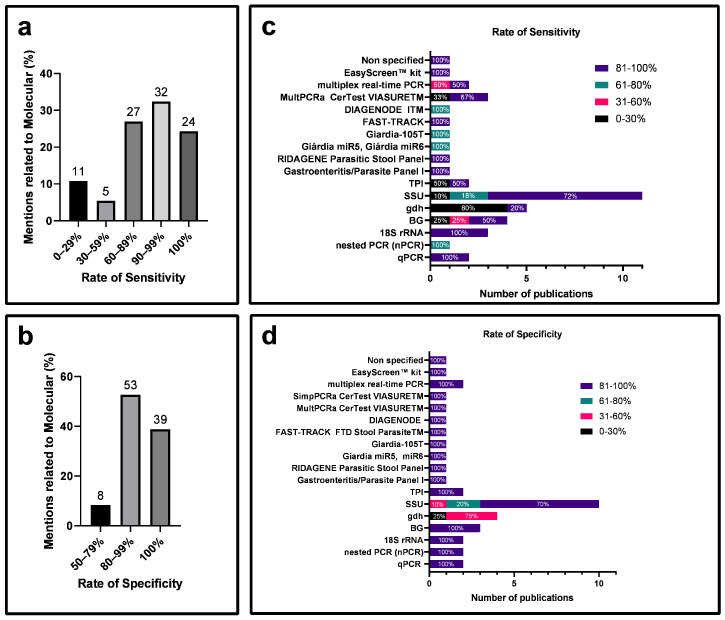
Classification of articles for the molecular diagnostics of giardiasis from selected sources. (**a**) Categorization of giardiasis molecular diagnostic articles based on sensitivity rates. (**b**) Categorization of giardiasis molecular diagnostic articles based on specificity rates. (**c**) Percentage representation of sensitivity rates for techniques employed in the molecular method. (**d**) Percentage representation of specificity rates for techniques utilized in the molecular method.

We compared the sensitivity (Figure 7a) and specificity (Figure 7b) of molecular methods. In terms of sensitivity, 32% of the articles reported rates of 90 to 99%, and 24% reported rates of 100% (Figure 7a). Regarding specificity, 53% of the reports presented rates between 80 and 99%, and specifically, 24% achieved 100% specificity. More specifically, 72% of the articles showed sensitivity between 81 to 100% (Figure 7c), and 70% demonstrated a specificity between 81 to 100% (Figure 7d). However, the GDH gene showed 80% of the reports with sensitivity between 0 and 30% (Figure 7c) and 100% of the reports with a specificity between 0 and 60% (Figure 7d).

## 4. Discussion

In 2004, the World Health Organization (WHO) included giardiasis in the group of the neglected disease initiative [3], underlining the significance of diagnosing this parasitic infection due to its association with abdominal discomfort, diarrhea, and other distressing symptoms. Swift and accurate diagnosis is particularly crucial in childhood, given the reported consequences like failure to thrive, which impedes physical and intellectual growth in children with chronic giardiasis [18]. The efficacy of a diagnostic or screening test can be appraised based on two parameters: specificity and sensitivity.

Specificity, as defined by Trevethan [19], is the likelihood of a test correctly identifying individuals who genuinely lack a disease among those guaranteed not to have it. It ensures the accurate exclusion of individuals without the condition. On the contrary, sensitivity refers to a test’s capacity to correctly identify positive cases among individuals suspected of having a disease, representing the likelihood of the test accurately detecting the presence of the disease in this specific group. In contrast, specificity aims to accurately identify negative cases, preventing the erroneous classification of healthy individuals as positive, thereby avoiding false negatives [19]. Over time, various techniques have emerged, including microscopic and immunological methods, and with the advent of molecular tools, PCR. This latter technique aims to optimize clinical outcomes for prompt pharmacological intervention, thereby preserving patient health [12]. Despite limited information on sensitivity and specificity in some articles (Supplementary Table S1), a comparative assessment of the reliability of microscopic, immunological, and molecular tests was feasible. Immunological and molecular methods exhibited sensitivity between 90 and 100%, surpassing the microscopic method. However, a higher percentage of articles demonstrated 100% specificity in the microscopic method (Figure 5a,b, Figure 6a,b and Figure 7a,b).

The prevalent microscopy tests in the papers are direct stool exams and the Ritchie method. Despite the availability of various diagnostic techniques, the direct wet mount stands out as a reliable method for diagnosing intestinal parasitic infections due to its unique ability to detect the motile trophozoite stage in soft-to-watery fecal specimens. However, challenges such as timely processing within 1 h and the recommendation for alternative methods like formal-ether sedimentation techniques, such as Ritchie, highlight the intricacies of practical application [20,21].

Methods employing microscopy for *Giardia* diagnosis are widely utilized in routine laboratory testing, offering advantages like simultaneous detection of multiple parasites, affordability, and ease of implementation. However, these methods often demonstrate low sensitivity due to the intermittent excretion of cysts in feces, necessitating multiple sample examinations for enhanced parasitological diagnosis efficiency. In contrast, immunoassays for *Giardia* antigen detection, especially ELISA, offer high sensitivity and specificity in giardiasis diagnosis [22].

While ELISAs boast commendable attributes, encompassing expeditiousness and sensitivity, they are not without noteworthy drawbacks, notably high costs and the imperative acquisition of supplementary equipment such as an ELISA reader. The immunochromatographic assays (ICAs) emerge as an alternative pathway, indicating a notable increase in their application in the field of immune methods, as depicted in Figure 4c. These assays, while adept at detecting antigens, stand out for their straightforward execution, eliminating the need for additional equipment. Immunochromatography, also known as lateral flow tests, seamlessly integrates the separation of sample molecules and reagents through capillary flow on a solid support, grounding its identification and detection mechanisms in the antigen–antibody immune reaction [23].

The realm of emerging molecular techniques, pivoting around the amplification of parasite DNA, prominently features the PCR technique. Quantitative polymerase chain reaction (qPCR)-based assays unfurl a promising vista for diagnosing infections, showcasing commendable sensitivity and specificity that enables the direct detection of *Giardia* DNA from fecal samples. It remains imperative to underscore that a negative result, in this context, does not categorically negate the presence of the parasite, as PCR inhibitors in feces may impede the amplification of DNA [22,23].

A discernible correlation surfaces between a higher incidence of giardiasis and countries in developmental stages [2,12]. This study substantiates this perspective, delineating that nations contributing significantly to the discourse on diagnostic methods for *Giardia* are predominantly in developmental phases. Noteworthy is the fact that despite the pervasive SARS-CoV-2 pandemic in 2020, the results underscore a substantial upswing in publications, accentuating the pivotal role of diagnosis. A meticulous analysis reveals an abundance of data linked to the diagnosis of giardiasis through microscopy, a consequence believed to stem from its stature as the standard tool for such diagnoses [10,24,25]. Fecal samples remain the focal point of sample analyses, despite select publications venturing into alternative sample types (Supplementary Figure S2).

Recent studies increasingly interweave microscopic diagnosis with immune and molecular techniques, with select works harmonizing all three methodologies concurrently [26,27,28]. Nevertheless, reports surface regarding diagnostic pitfalls associated with immunological and molecular techniques due to detection issues [29,30], thereby underscoring the enduring necessity of incorporating microscopic techniques.

In the realm where microscopy is regarded as the standard diagnostic test for giardiasis, it is imperative to elucidate inherent disadvantages. The work by Paulos and collaborators [31] sheds light on the non-species-specific nature of these methods, rendering it impossible in epidemiological research to discern variations of strains and different species. Additionally, the characterization of the method as “laborious” by the authors emphasizes the considerable exertion demanded of professionals, especially when confronted with numerous samples. This laborious nature arises from the intricate processes inherent to the chosen method, where each sample mandates individual scrutiny by the professional [31]. Literature corroborates that the experience and proficiency of the professional executing the technique are pivotal, necessitating the identification of cysts, trophozoites, and eggs within biological samples laden with extraneous material that could potentially obfuscate microorganism structures [25,26,27,28,29,30,31,32,33,34].

An additional critical facet in achieving a more dependable result involves guiding patients to collect multiple samples on alternate days, considering the quantitative variation in cyst elimination during infection. Thus, soliciting patients to procure three samples, with an interval of at least three days between each, escalates sensitivity to an impressive 94%, in contrast to the 50–70% sensitivity associated with analyzing a singular sample [10,12,24]. The crux of the diagnostic challenge does not rest in the technique but in the clinicians, who oftentimes overlook the need for a parasitological examination due to the overlap of clinical symptoms with other diseases, rendering the diagnosis inconspicuous [10].

Light microscopy methods, renowned for their cost-effectiveness [32,33,34,35,36], harbor the added advantage of diagnosing a spectrum of intestinal parasites beyond giardiasis, including ascariasis, amoebiasis, taeniasis, and hookworm [12,34,36]. The Ritchie method emerges as the favored approach according to the data gleaned from selected articles. Concentration methods, deemed more judicious, significantly elevate the probability of securing a dependable diagnosis, given their heightened sensitivity compared to direct examination [10,35,36,37]. Noteworthy findings by Doni and collaborators [38] underscore that, when subjected to comparative analysis against direct examination, the latter yielded a 42% false-negative rate, while the concentration method exhibited a mere 14% incidence of false-negative reports. Ritchie’s method, in particular, exhibits superior sensitivity relative to alternative methodologies [39,40].

ELISA tests are extolled for their heightened sensitivity compared to microscopic methods, conferring up to a 30% greater likelihood of detecting *G. intestinalis* in fecal samples. This aligns seamlessly with the data corroborated in this study [41,42,43,44]. In addition to heightened sensitivity and specificity, the expeditious nature of the method (yielding results in 10 to 15 min) enhances its efficacy for epidemiological or endemic research [25,41,43,44,45]. Notably, ELISA serves as a versatile methodology capable of simultaneous diagnosis of other intestinal parasites through fecal material analysis [42,43]. Within the corpus of articles scrutinized, commercial kits emerge as the predominant choice for respective tests. Unfortunately, the precise antigens utilized for the diagnosis of giardiasis by ELISA remain shrouded in secrecy, presumably due to proprietary considerations.

PCR, though the most recent entrant among diagnostic techniques, aligns itself with superior sensitivity and specificity among all available methodologies for diagnosing giardiasis [44]. The utilization of multiple gene loci further augments sensitivity, mitigating the risk of false results. While primers targeting beta-giardin are deemed particularly sensitive, it is acknowledged that the complex nature of stool samples may engender gene material degradation, thereby elevating the likelihood of false negatives [46]. Notwithstanding PCR’s classification as the preeminent methodology for giardiasis diagnosis, practical challenges loom large, principally attributed to its cost [46]. Offering PCR diagnostic services poses an even more formidable challenge, particularly in developing countries where infrastructural support for such robust techniques is often lacking due to equipment constraints [25]. According to Soares and collaborators [35], the prohibitive cost associated with immunological and molecular methods emerges as the primary limiting factor, casting microscopic methods as the preeminent choice.

Reflecting on the data at hand, it becomes apparent that, concerning the diagnosis of giardiasis, the majority of published works gravitate towards microscopy, followed by PCR and subsequently ELISA. This hierarchy is intrinsically tied to the economic aspect; microscopic examination of stool samples stands out as the most cost-effective method, obviating the need for large-scale equipment such as an ELISA plate reader or a PCR thermal cycler, not to mention the employment of more sophisticated reagents. Armed with nothing more than a microscope, a sedimentation cup, a dye (lugol), a slide, and a coverslip, one can adeptly detect a spectrum of parasites, including *G. intestinalis*. In the calculus of cost and benefits, while ELISA and PCR methods offer heightened sensitivity and specificity, their prohibitive costs, particularly in the case of PCR, emerge as the principal impediment. Contemplating the establishment of a diagnostic laboratory and the integration of more sensitive methods necessitates meticulous consideration of costs, wherein the substitution of a cost-effective, broadly applicable, and reliable technique with a more sensitive variant incurring substantially higher costs poses a formidable economic challenge.

## 5. Conclusions

Hence, derived from the outcomes delineated in this study, the adoption of microscopic concentration methods is recommended as the primary modality within clinical settings, given its manifold advantages. With respect to mitigating false-negative outcomes, it is advisable to guide patients to adhere to the aforementioned sample collection protocol, thereby augmenting sensitivity and minimizing the probability of inconclusive results. In instances where a negative result persists amid indicative symptoms, the implementation of the ELISA method is warranted, given its heightened sensitivity. Notably, the utilization of PCR would be more judicious in characterizing resilient species and strains, albeit hindered by its elevated cost.

## Data Availability

Not applicable.

## Supplementary Materials

The following supporting information can be downloaded at: https://www.mdpi.com/article/10.3390/diagnostics14040364/s1, Figure S1: Analysis of the most frequently mentioned words in the abstracts of selected articles using Word Clouds; Figure S2: Analysis of sample types and kits employed in each method is illustrated; Table S1: Analysis of a total of 163 articles addressing diagnostic methods for giardiasis; Table S2: Quantification of types of diagnostic techniques employed in each country; Table S3: PRISMA checklist.

## References

[1] Squire S.A., Ryan U. (2017). *Cryptosporidium* and *Giardia* in Africa: Current and future challenges. Parasite Vectors.

[2] Adam R.D. (2001). Biology of *Giardia lamblia*. Clin. Microbiol. Rev..

[3] Savioli L., Smith H., Thompson A. (2006). *Giardia* and *Cryptosporidium* join the ‘Neglected Diseases Initiative’. Trends Parasitol..

[4] Coffey C.M., Collier S.A., Gleason M.E., Yoder J.S., Kirk M.D., Richardson A.M., Fullerton K.E., Benedict K.M. (2021). Evolving Epidemiology of Reported Giardiasis Cases in the United States, 1995–2016. Clin. Infect. Dis..

[5] Kirk M.D., Pires S.M., Black R.E., Caipo M., Crump J.A., Devleesschauwer B., Döpfer D., Fazil A., Fischer-Walker C.L., Hald T. (2015). World Health Organization Estimates of the Global and Regional Disease Burden of 22 Foodborne Bacterial, Protozoal, and Viral Diseases, 2010: A Data Synthesis. PLoS Med..

[6] Lanata C.F., Fischer-Walker C.L., Olascoaga A.C., Torres C.X., Aryee M.J., Black R.E. (2013). Global causes of diarrheal disease mortality in children <5 years of age: A systematic review. PLoS ONE.

[7] Botero-Garcés J.H., García-Montoya G.M., Grisales-Patiño D., Aguirre-Acevedo D.C., Álvarez-Uribe M.C. (2009). *Giardia intestinalis* and nutritional status in children participating in the complementary nutrition program, Antioquia, Colombia, May to October. Rev. Inst. Med. Trop. São Paulo.

[8] Busatti H.G.N.O., Santos J.F.G., Gomes M.A. (2009). The old and new therapeutic approaches to the treatment of giardiasis: Where are we?. Biologics.

[9] Ankarklev J., Jerlstrom-Hultqvist J., Ringqvist E., Troell K., Svard S.G. (2010). Behind the smile: Cell biology and disease mechanisms of *Giardia* species. Nat. Rev. Microbiol..

[10] Escobedo A.A., Almirall P., Hanevik K., Cimerman S., Rodríguez-Morales A.J., Almanza C., Auza-Santivañez J. (2018). Giardiasis: A diagnosis that should be considered regardless of the setting. Epidemiol. Infect..

[11] Cantey P.T., Roy S., Lee B., Cronquist A., Smith K., Liang J., Beach M.J. (2011). Study of nonoutbreak giardiasis: Novel findings and implications for research. Am. J. Med..

[12] Neves D.P., Neves D.P., Viana S.G.F., Sogayar M.I.T.L. (2016). Giardia. Parasitologia Humana.

[13] Guy R.A., Xiao C., Horgen P.A. (2004). Real-time PCR assay for detection and genotype differentiation of *Giardia lamblia* in stool specimens. J. Clin. Microbiol..

[14] Berne A.C., Vieira J.N., de Avila L.F., da Costa L.F., Villela M.M., Berne M.E.A., Scaini C.J. (2014). *Giardia lamblia*: DIAGNÓSTICO COM O EMPREGO DE MÉTODOS Microscópicos E Enzyme-linked Immunosorbent Assay (ELISA). Rev. Patol. Trop..

[15] Van Den Brandhof W.E., Bartelds A.I., Koopmans M.P., van Duynhoven Y.T. (2006). General practitioner practices in requesting laboratory tests for patients with gastroenteritis in the Netherlands, 2001–2002. BMC Fam. Pract..

[16] Widmer G., Carmena D., Kváč M., Chalmers R.M., Kissinger J.C., Xiao L., Sateriale A., Striepen B., Laurent F., Lacroix-Lamandé S. (2020). Update on *Cryptosporidium* spp.: Highlights from the Seventh International Giardia and *Cryptosporidium* Conference. Parasite.

[17] Page M.J., McKenzie J.E., Bossuyt P.M., Boutron I., Hoffmann T.C., Mulrow C.D., Shamseer L., Tetzlaff J.M., Akl E.A., Brennan S.E. (2021). The PRISMA 2020 Statement: An Updated Guideline for Reporting Systematic Reviews. BMJ.

[18] Robertson L.J., Hanevik K., Escobedo A.A., Mørch K., Langeland N. (2010). Giardiasis—Why do the symptoms sometimes never stop?. Trends Parasitol..

[19] Trevethan R. (2017). Sensitivity, Specificity, and Predictive Values: Foundations, Pliabilities, and Pitfalls in Research and Practice. Front. Public Health.

[20] Demeke G., Fenta A., Dilnessa T. (2021). Evaluation of Wet Mount and Concentration Techniques of Stool Examination for Intestinal Parasites Identification at Debre Markos Comprehensive Specialized Hospital, Ethiopia. Infect. Drug Resist..

[21] Anécimo R.S., Tonani K.A.A., Fregonesi B.M., Mariano A.P., Ferrassino M.D.B., Trevilato T.M.B., Rodrigues R.B., Segura-Muñoz S.I. (2012). Adaptation of Ritchie’s Method for Parasites Diagnosing with Minimization of Chemical Products. Interdiscip. Perspect. Infect. Dis..

[22] Silva R.K.N.R., Pacheco F.T.F., Martins A.S., Menezes J.F., Costa-Ribeiro H., Ribeiro T.C.M., Mattos Â.P., Oliveira R.R., Soares N.M., Teixeira M.C.A. (2016). Performance of microscopy and ELISA for diagnosing *Giardia duodenalis* infection in different pediatric groups. Parasitol. Int..

[23] Banisch D.M., El-Badry A., Klinnert J.V., Ignatius R., El-Dib N. (2015). Simultaneous detection of Entamoeba histolytica/dispar, *Giardia duodenalis* and cryptosporidia by immunochromatographic assay in stool samples from patients living in the Greater Cairo Region, Egypt. World J. Microbiol. Biotechnol..

[24] Uchôa F.F.M., Sudré A.P., Macieira D.B., Almosny N.R.P. (2017). The influence of serial fecal sampling on the diagnosis of giardiasis in humans, dogs, and cats. Rev. Inst. Med. Trop. São Paulo.

[25] Alhamd S. (2020). Giardiasis in the UK: Current Prevalence, Diagnosis and Treatment. Xi’an Jianzhu Keji Daxue Xuebao.

[26] Kaminsky R.G., García J.A. (2022). Evaluación de pruebas inmunológicas en el diagnóstico de *Giardia duodenalis* y *Cryptosporidium* spp., Honduras. Rev. Méd. Hondureña.

[27] Fantinatti M., Cascais-Figueredo T., Austriaco-Teixeira P., Carvalho-Costa F.A., Da-Cruz A.M. (2023). *Giardia lamblia*-infected preschoolers present growth delays independent of the assemblage A, B or E. Mem. Inst. Oswaldo Cruz.

[28] Alharbi A., Toulah F.H., Wakid M.H., Azhar E., Farraj S., Mirza A.A. (2020). Detection of *Giardia lamblia* by Microscopic Examination, Rapid Chromatographic Immunoassay Test, and Molecular Technique. Cureus.

[29] Schuurman T., Lankamp P., van Belkum A., Kooistra-Smid M., van Zwet A. (2007). Comparison of microscopy, real-time PCR and a rapid immunoassay for the detection of *Giardia lamblia* in human stool specimens. Clin. Microbiol. Infect..

[30] Hanevik K., Hausken T., Morken M.H., Strand E.A., Mørch K., Coll P., Helgeland L., Langeland N. (2007). Persisting symptoms and duodenal inflammation related to *Giardia duodenalis* infection. J. Infect..

[31] Paulos S., Mateo M., de Lucio A., Hernández-de Mingo M., Bailo B., Saugar J.M., Cardona G.A., Fuentes I., Mateo M., Carmena D. (2016). Evaluation of five commercial methods for the extraction and purification of DNA from human faecal samples for downstream molecular detection of the enteric protozoan parasites *Cryptosporidium* spp., *Giardia duodenalis*, and *Entamoeba* spp.. J. Microbiol. Methods.

[32] El-Nahas H.A., Salem D.A., El-Henawy A.A., El-Nimr H.I., Abdel-Ghaffar H.A., El-Meadawy A.M. (2013). *Giardia* diagnostic methods in human fecal samples: A comparative study. Cytom. B Clin. Cytom..

[33] Sadaka H.A., Gaafar M.R., Mady R.F., Hezema N.N. (2015). Evaluation of ImmunoCard STAT test and ELISA versus light microscopy in diagnosis of giardiasis and cryptosporidiosis. Parasitol. Res..

[34] Elswaifi S.F., Palmieri J.R., El-Tantawy N., El-Hussiny M., Besheer T., Abohashem E. (2016). Comparison of microscopic and immunoassay examination in the diagnosis of intestinal protozoa of humans in Mansoura, Egypt. J. Parasit. Dis..

[35] Soares R., Tasca T. (2016). Giardiasis: An update review on sensitivity and specificity of methods for laboratorial diagnosis. J. Microbiol. Methods.

[36] EL-Lessy F.M., Zekry K.M., Hasan A.T., Aly I.R. (2019). Evaluation of nano-graphene based sandwich and dot-elisa as promising techniques for diagnosis of human intestinal giardiasis. J. Egypt. Soc. Parasitol..

[37] Madbouly N.A., Farid A., El-Badry A.A., El-Amir A.M. (2016). Immune-molecular identification of *Giardia intestinalis* in diarrhoeal children: Comparison of three diagnostic methods. J. Egypt. Soc. Parasitol..

[38] Doni N.Y., Zeyrek F.Y., Gürses G., Tümer S. (2013). Comparison of direct microcopy and antigen casette tests for the detection of *Giardia* and *Cryptosporidium*. Turk. Parazitol. Derg..

[39] Jahan N., Khatoon R., Ahmad S. (2014). A Comparison of Microscopy and Enzyme Linked Immunosorbent Assay for Diagnosis of *Giardia lamblia* in Human Faecal Specimens. J. Clin. Diagn. Res..

[40] Hooshyar H., Rostamkhani P., Arbabi M., Delavari M. (2019). *Giardia lamblia* infection: Review of current diagnostic strategies. Gastroenterol. Hepatol. Bed Bench.

[41] Minak J., Kabir M., Mahmud I., Liu Y., Liu L., Haque R., Petri W.A. (2012). Evaluation of rapid antigen point-of-care tests for detection of *Giardia* and *Cryptosporidium* species in human fecal specimens. J. Clin. Microbiol..

[42] Heyworth M.F. (2014). Diagnostic testing for *Giardia* infections. Trans. R. Soc. Trop. Med. Hyg..

[43] Gaafar M.R. (2011). Evaluation of enzyme immunoassay techniques for diagnosis of the most common intestinal protozoa in fecal samples. Int. J. Infect. Dis..

[44] Nooshadokht M., Kalantari-Khandani B., Sharifi I., Kamyabi H., Liyanage N.P.M., Lagenaur L.A., Kagnoff M.F., Singer S.M., Babaei Z., Solaymani-Mohammadi S. (2017). Stool antigen immunodetection for diagnosis of *Giardia duodenalis* infection in human subjects with HIV and cancer. J. Microbiol. Methods.

[45] Singhal S., Mittal V., Khare V., Singh Y.I. (2015). Comparative analysis of enzyme-linked immunosorbent assay and direct microscopy for the diagnosis of *Giardia intestinalis* in fecal samples. Indian. J. Pathol. Microbiol..

[46] Uchôa F.F.M., Sudré A.P., Campos S.D.E., Almosny N.R.P. (2018). Assessment of the diagnostic performance of four methods for the detection of *Giardia duodenalis* in fecal samples from human, canine and feline carriers. J. Microbiol. Methods.

