# Supplementary material for: Systematic Review of Diagnostic Approaches for Human Giardiasis: Unveiling Optimal Strategies
Source: Diagnostics (Basel). 2024 Feb 7;14(4):364. doi: 10.3390/diagnostics14040364 (PMC10887752; doi:10.3390/diagnostics14040364)
Supplement: Supplementary file 1 [file diagnostics-14-00364-s001.zip › diagnostics-2839715-supplementary.pdf]

Supplementary Figure Legend

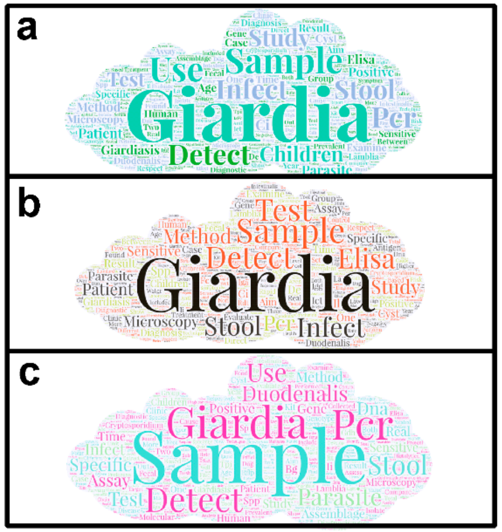

Supplementary Figure S1. Analysis of the most frequently mentioned words in the abstracts of selected articles using Word Clouds. (**Fig. a**) Displays the most cited words in abstracts related to microscopic diagnostic techniques. (**Fig. b**) Focuses on the most common words in abstracts related to immunoassay diagnostic techniques. (**Fig. c**) Exhibits the most cited words in abstracts of articles related to diagnostic techniques by molecular methods.

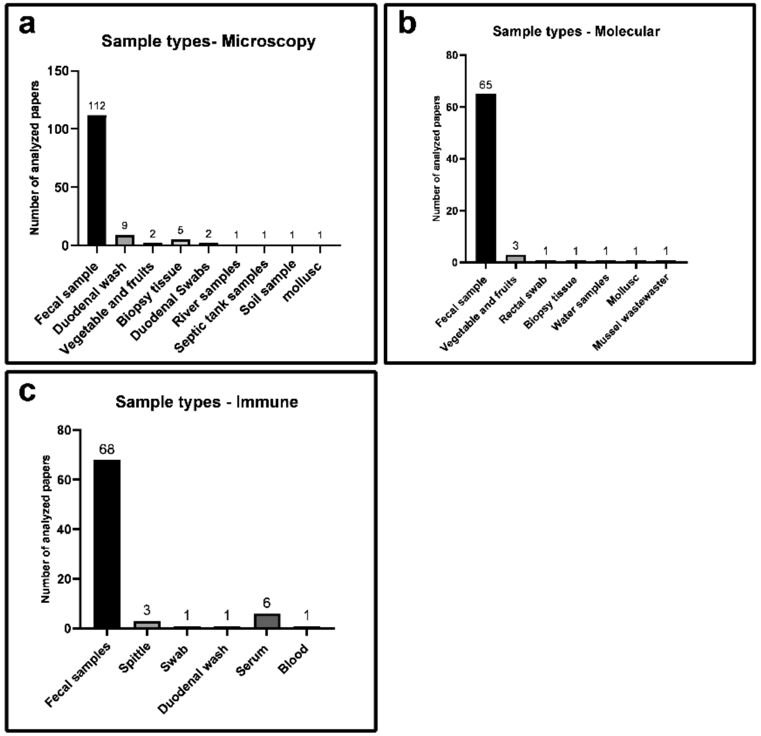

Supplementary Figure S2. Analysis of sample types and kits employed in each method is illustrated. (FIG. a) Highlights the sample types utilized in the microscopy analysis of the papers. (FIG. b) Focuses on the sample types employed in the molecular analysis of the papers. (FIG. c) Outlines the sample types utilized in the immune assays of the papers.

Supplementary Table S1 – Analysis of total 163 articles addressing diagnostic methods for giardiasis.

Table S1

					Microscopy	Immune	Molecular							
Title	Year	Country	Sample	Method	Dye / target	Sens. (%)	Spec (%)	Kits / Antigen	Sens. (%)	Spec (%)	Kit / Targets	Sens. (%)	Spec (%)	Ref. DOI
**RESERVOIRS OF GIARDIA SPP. IN SOUTHWESTERN ALBERTA**	1984	Canada	Fecal sample	Microscopy<br>(Roberts-Thomson)	-	-	-	-	-	-	-	-	-	10.7589/0090-3558-20.4.279
**Enzyme-linked immunosorbent assay for the detection of anti-Giardia specific immunoglobulin G in filter paper blood samples**	1993	Saudi Arabia	serum	ELISA	-	-	-	IgG anti-Giardia	91	95	-	-	-	10.1016/0035-9203(93)90412-j
**Diagnosis of Giardia duodenalis infection in Bangladeshi infants: faecal antigen capture ELISA**	1993	Banglad esh	Fecal sample	Microscopy<br>(Direct / Ritchie)<br>ELISA	-	-	-	IgG anti-Giardia	94	98	-	-	-	10.1016/0035-9203(93)90025-l
**Transactions of the Royal Society of Tropical Medicine and Hygiene**	1993	Australia	Fecal sample	Microscopy<br>(Faust)<br>ELISA	Sargeaunt	-	-	CELISA	100	-	-	-	-	10.1016/0035-9203(93)90414-l
**Evaluation of a commercially available ELISA for detection ofGiardia lamblia antigen in faeces: Preliminary results in unconventional samples**	1995	Italy	Fecal sample	Microscopy<br>(Ritchie)	-	-	-	CELISA detection kit	-	-	-	-	-	10.1007/bf01720307

				ELISA										
**Laboratory Diagnosis for Giardia Lamblia Infection: A Comparison of Microscopy, Coprodiagnosis and Serology**	1997	Canada	Fecal sample / serum	Microscopy<br>(Ritchie)<br>Sandwich ELISA / ELISA / Western Blot	hematoxylin	-	-	kit ProSpecT / IgG; IgM / IgG; IgM and IgA	87 / 50 / 71	96 / 56 / 78	-	-	-	10.1155/1997/270179
**Intestinal amoebiasis, giardiasis and geohelminthiases: their association with other intestinal parasites and reported intestinal symptoms**	1999	Costa do Marfim	Fecal sample	Microscopy<br>(Ritchie)	-	-	-	-	-	-	-	-	-	10.1016/s0035-9203(99)90287-0
**Serodiagnosis of giardiasis: identiflcation of immunoglobulin G anti-Giardia duodenalis in sera by ELISA**	2001	Colombi a	Fecal sample / serum	Microscopy<br>ELISA	Lugol	-	-	IgG anti-Giardia duodenalis	98	95	-	-	-	10.7705/biomedica.v21i3 .1112
**Occurrence of Cryptosporidium oocysts and Giardia cysts in raw water from the Atibaia river, Campinas, Brazil**	2001	Brazil	Water sample	Microscopy<br>(Immunofluorescenc e)<br>(Direct / Ritchie)	Merifluor	-	-	-	-	-	-	-	-	10.1590/s0036-46652001000200011
**Detection of Giardia duodenalis antigen in coprolites using a commercially available enzyme-linked immunosorbent assay**	2022	Brazil	Fecal sample	Microscopy<br>Lutz / Faust<br>ELISA	-	-	-	ProSnecT *Giardia*	-	-	-	-	-	10.1016/s0035-9203(02)90337-8

**Detection of Giardia duodenalis antigen in human fecal eluates by enzyme-linked immunosorbent assay using polyclonal antibodies**	2022	Colombia	Fecal sample	Microscopy<br>(Direct / Ritchie)<br>ELISA	-	-	-	IgG anti-*Giardia*	100	95	-	-	-	10.1590/s0074-02762002000800018
**Identification of Immunoreactive Proteins during Acute Human Giardiasis**	2003	Sweden	serum	Microscopy<br>(Immunofluorescence indirect (IFL))<br>Western Blot	-	-	-	IgG e IgA	-	-	-	-	-	10.1086/375356
**Secretory IgA Antibody Responses in Venezuelan Children Infected with Giardia duodenalis**	2004	Venezuela	Fecal sample / serum / Spittle	Microscopy<br>(Direct)<br>ELISA	-	-	-	Secretory IgA antibodies (sIgA)	74	95	-	-	-	10.1093/tropej/50.2.68
**Albendazole versus metronidazole treatment of adult giardiasis: An open randomized clinical study**	2004	Turkey	Fecal sample	Microscopy<br>(Direct)	-	-	-	-	-	-	-	-	-	10.3748/wjg.v10.i8.1215
**Enzyme-linked immunosorbent assay (ELISA) immunoassaying versus microscopy: advantages and drawbacks for diagnosing giardiasis**	2005	Brazil	Fecal sample	Microscopy<br>(Hoffman / Faust)<br>ELISA	-	-	-	Prospect ELISA	-	-	-	-	-	10.1590/s1516-31802005000600006

**Comparison of Two Target Genes for Detection and Genotyping of Giardia lamblia in Human Feces by PCR and PCR-Restriction Fragment Length Polymorphism**	2005	France	Fecal sample	Microscopy<br>(Ritchie / Immunofluorescence)<br>PCR	PI / mAb-FITC	-	-	-	-	-	tpi	-	-	10.1128/jcm.43.12.5940-5944.2005
**Dientamoeba fragilis is more prevalent than Giardia duodenalis in children and adults attending a day care centre in Central Italy**	2005	Italy	Fecal sample	Microscopy<br>(Direct)	-	-	-	-	-	-	-	-	-	10.1051/parasite/2005122165
**Giardia lamblia infection in patients with irritable bowel syndrome and dyspepsia: A prospective study**	2006	Italy	Fecal sample / duodenal biopsy	Microscopy<br>(Direct / Ritchie / Immunofluorescence)<br>ELISA / Immunochromatographic	giemsa / trichrome / acridine orange / hematoxylin-eosina / Merifluor	22	100	Xpect / Prospect	-	-	-	-	-	10.3748/wjg.v12.i12.1941
**A large community outbreak of waterborne giardiasis- delayed detection in a non-endemic urban area**	2006	Norway	Fecal sample	Microscopy<br>-<br>Immunochromatography	-	-	-	ImmunoCard STAT!	-	-	-	-	-	10.1186/1471-2458-6-141
**Application of Genotyping during an Extensive Outbreak of Waterborne Giardiasis in Bergen,**	2006	Norway	Fecal sample / septic tank / Amostra de solo	Microscopy	Aqua-Glo	-	-	ImmunoCard STAT!	-	-	-	-	-	10.1128/aem.72.3.2212-2217.2006

**Norway, during Autumn and Winter 2004†**														
				(Ritchie / Immunofluorescence)										
				Immunochromatography										
**Occurrence of enteroparasites in day care centers in Botucatu (São Paulo State, Brazil) with emphasis on Cryptosporidium sp., Giardia duodenalis and Enterobius vermicularis**	2006	Brazil	Fecal sample	Microscopy	-	-	-	-	-	-	-	-	-	10.1590/s0036-46652006000500006
				(Hoffman / Faust / Ritchie)										
**Comparison of microscopy, real-time PCR and a rapid immunoassay for the detection of Giardia lamblia in human stool specimens**	2007	Netherlands	Fecal sample	Microscopy	Lugol / Iodo + clorazol	99	97	ImmunoCard STAT!	98	100	SSU rRNA	100	92	10.1111/j.1469-0691.2007.01836.x
				(Ridley / Direct)										
				Immunochromatography										
				qPCR										
**Persisting symptoms and duodenal inflammation related to Giardia duodenalis infection**	2007	Norway	Fecal sample / duodenal fluid	Microscopy	-	-	-	ImmunoCard STAT!	-	-	-	-	-	10.1016/j.jinf.2007.09.004
				(Ritchie)										
				Immunochromatography										
**Molecular characterisation of Giardia isolates from clinical infections following a waterborne outbreak**	2007	Norway	Fecal sample	Microscopy	-	-	-	ImmunoCard STAT!	-	-	-	-	-	10.1016/j.jinf.2007.02.001
				(Ritchie)										

				Immunochromatography										
**Sensitivity of a Giardia antigen test in persistent giardiasis following an extensive outbreak**	2008	Norway	Fecal sample	Microscopy<br>(Ritchie)<br>Immunochromatography	-	-	-	ImmunoCard STAT!	60	96	-	-	-	10.1111/j.1469-0691.2008.02078.x
**Role of gastric brush cytology in the diagnosis of giardiasis**	2008	India	Endoscopic brush smears (EBS)	Microscopy<br>-	Pap smear / Giemsa	-	-	-	-	-	-	-	-	10.4103/0970-9371.42443
**Giardia duodenalis infection and anthropometric status in preschoolers in Salvador, Bahia State, Brazil**	2008	Brazil	Fecal sample	Microscopy<br>(Sedimentação espontânea)	-	-	-	-	-	-	-	-	-	10.1590/s0102-311x2008000700007
**Evaluation of a real-time polymerase chain reaction assay for the laboratory diagnosis of giardiasis**	2009	Italy	Fecal sample	Microscopy<br>(Ritchie / Immunofluorescence)<br>Immunochromatography<br>qPCR	Merifluor	86	100	ImmunoCard STAT!	86	100	SSU rRNA	100	100	10.1016/j.diagmicrobio.2009.10.004
**Transmission cycles of Giardia duodenalis in dogs and humans in Temple communities in Bangkok—A critical evaluation of its prevalence using three diagnostic tests in the field in the absence of a gold standard**	2009	Thailand	Fecal sample	Microscopy<br>(Faust / Roberts-Thomson /	Aqua-Glo	26 / 61	99 / 94	CELISA	-	-	SSU rRNA	97	56	10.1016/j.actatropica.2009.03.006

				Immunofluorescence)<br>ELISA<br>nPCR										
**IFN-gamma, IL-5, IL-6 and IgE in patients infected with Giardia intestinalis.**	2009	Poland	Fecal sample / duodenal fluid	Microscopy<br>-<br>ELISA	-	-	-	GSA-65	-	-	-	-	-	10.2478/v10042-009-0013-3
**Prevalence of recurring symptoms after infection with Giardia lamblia in a non-endemic area**	2009	Norway	Fecal sample	Immunochromatography	-	-	-	ImmunoCard STAT!	-	-	-	-	-	10.1080/02813430802602393
**Real-Time Pcr/rflp Assay to Detect Giardia intestinalis Genotypes in Human Isolates with Diarrhea in Egypt**	2009	Egypt	Fecal sample	Microscopy<br>(Direct)<br>PCR	Lugol	-	-	-	-	-	tpi	-	-	10.1645/ge-1670.1
**Giardiasis in kindergartens: prevalence study in Berlin, Germany, 2006**	2009	Germany	Fecal sample	Microscopy<br>(Immunofluorescence / -)	-	-	-	-	-	-	-	-	-	10.1007/s00436-009-1438-5
**Inducible nitric oxide synthase in duodenum of children with Giardia lamblia infection.**	2010	Poland	Fecal sample / duodenal fluid	Microscopy<br>-<br>ELISA	-	-	-	Crypto/Giardia Duo Strip	-	-	-	-	-	10.2478/v10042-008-0111-7
**A case of giardiasis expressing severe systemic symptoms and marked hypereosinophilia**	2010	Japan	Fecal sample	Microscopy<br>-	-	-	-	-	-	-	-	-	-	10.1016/j.parint.2010.06.006
**Impact of Giardia lamblia on Growth, Serum Levels of Zinc, Copper, and**	2010	Egypt	Fecal sample	Microscopy	Lugol / -	-	-	-	-	-	-	-	-	10.1007/s12011-010-8673-6

**Iron in Egyptian Children**				-										
**Assessment of the levels of nitric oxide (NO) and cytokines (IL-5, IL-6, IL-13, TNF, IFN-gamma) in giardiosis**	2011	Poland	Fecal sample	ELISA	-	-	-	GSA-65	-	-	-	-	-	10.5603/fhc.2011.0039
**Identification and molecular characterization of Cryptosporidium and Giardia in children and cattle populations from the province of Álava, North of Spain**	2011	Spain	Fecal sample	Microscopy (Ritchie)<br>ELISA / Immunochromatographic	-	-	-	CpAg-ELISA / Operon	83 / 75	97 / 99	-	-	-	10.1016/j.scitotenv.2011.09.076
**Evaluación de dos métodos inmunocromatográficos comerciales para el diagnóstico rápido de Giardia duodenalis y Cryptosporidium spp. en muestras de heces**	2011	Spain	Fecal sample	Microscopy (Ritchie)<br>Immunochromatographic<br>PCR	Lugol	-	-	CerTes / Operon	97 / 97	100 / 95	SSU rRNA	-	-	10.1016/j.eimc.2010.09.005
**Hospitalization of Cuban children for giardiasis: a retrospective study in a paediatric hospital in Havana**	2011	Cuba	Fecal sample / duodenal fluid	Microscopy (Direct)	-	-	-	-	-	-	-	-	-	10.1179/136485911x12899838413420
**Investigating Dyspepsia in Clinical Practice - A Trap for Giardia**	2011	Slovakia	duodenal fluid	Microscopy (Direct)	-	-	-	-	-	-	-	-	-	10.2478/v10201-011-0004-x

**Diagnosis of Giardia infections by PCR-based methods in children of an endemic area**	2011	Brazil	Fecal sample	Microscopy<br>(Faust / kit TF-Test®)<br>Semi-nestedPCR / nPCR	-	99 / 79	-	-	-	-	gdh / tpi	-	-	10.1590/s1678-91992011000200012
**Evaluation of a PCR protocol for sensitive detection of Giardia intestinalis in human faeces**	2011	Australia	Fecal sample	PCR	-	-	-	-	-	-	18S rRNA / gdh			10.1007/s00436-011-2565-3
**Caregiver perspectives for the prevention, diagnosis and treatment of childhood giardiasis in Havana City, Cuba. A qualitative study**	2011	Cuba	Fecal sample / duodenal fluid / duodenal biopsy	-	-	-	-	-	-	-	-	-	-	10.1016/j.actatropica.2011.04.014
**Spittle and sera IgA and IgG in Egyptian Giardia-infected children**	2012	Egypt	Fecal sample / Spittle / blood	Microscopy<br>(Direct)<br>ELISA	Lugol / -	-	83	IgG / IgA	77	95	-	-	-	10.1007/s00436-012-2869-y
**Prevalence and risk factors for Giardia duodenalis infection among children: A case study in Portugal**	2012	Portugal	Fecal sample	Microscopy<br>(Direct)<br>ELISA	-	-	-	Ridascreen	-	-	-	-	-	10.1186/1756-3305-5-22
**Evaluation of an immunochromatographic dip strip test for simultaneous detection of Cryptosporidium spp, Giardia duodenalis, and Entamoeba histolytica antigens**	2012	Spain	Fecal sample	Microscopy	-	-	-	RidaQuick / Ridascreen	96 / 93	99 / 97	tpi	-	-	10.1007/s10096-012-1544-7

**in human faecal samples**				(Ritchie / Direct)<br>Immunochromatographic / ELISA<br>PCR										
**Giardia diagnostic methods in human fecal samples: A comparative study**	2012	Egypt	Fecal sample	Microscopy<br>(Ritchie / Immunofluorescence)<br>Cytometry	Giardia-Cel / -	76	100	Giardia-a-Glo	73	100	-	-	-	10.1002/cyto.b.21048
**Real-time PCR: Benefits for Detection of Mild and Asymptomatic Giardia Infections**	2012	Thailand	Fecal sample	Microscopy<br>-<br>qPCR	Lugol	-	-	-	-	-	SSU rRNA	-	-	10.2149/tmh.2012-08
**Molecular Epidemiology of Cryptosporidium and Giardia in Humans on Prince Edward Island, Canada: Evidence of Zoonotic Transmission From Cattle**	2012	Canada	Fecal sample	Microscopy<br>(Immunofluorescence)	Crypt-a-glo / Giardi-a-glo	-	-	-	-	-	-	-	-	10.1111/j.1863-2378.2012.01474.x
**Stool sample storage conditions for the preservation of Giardia intestinalis DNA**	2012	Turkey	Fecal sample	PCR	-	-	-	-	-	-	bga	-	-	10.1590/s0074-02762012000800001
**Rapid identification of Giardia duodenalis assemblages in NSW using terminal-restriction fragment**	2012	Australia	Fecal sample	PCR	-	-	-	-	-	-	gdh	-	-	10.1017/s0031182012000388

**length polymorphism**														
**Molecular characterizations of Cryptosporidium, Giardia, and Enterocytozoon in humans in Kaduna State, Nigeria**	2012	Nigeria	Fecal sample	nPCR	-	-	-	-	-	-	tpi	-	-	10.1016/j.exppara.2012.05.011
**Intestinal parasitic infections: high prevalence of Giardia intestinalis in children living in an orphanage compared with hill-tribe children as detected by microscopy and ELISA**	2013	Thailand	Fecal sample / Scotch tape	Microscopy (Ritchie)<br>ELISA	-	-	-	Giardia Antigen (Ref. 6005, Generic Assay)	72	91	-	-	-	10.5372/1905-7415.0706.250
**Detection of Giardia lamblia stool samples: a comparison of two enzyme-linked immunosorbent assays**	2013	Germany	Fecal sample	Microscopy (Direct / Immunofluorescence)<br>ELISA<br>PCR	Iron-hematoxylin	-	-	Ridascreen / Serazym	72 / 93	100 / 100	-	85	100	10.12688/f1000research.2-39.v1
**DETECTION OF GIARDIA LAMBLIA, CRYPTOSPORIDIUM SPP. AND ENTAMOEBA HISTOLYTICA IN CLINICAL STOOL SAMPLES BY USING MULTIPLEX REAL-TIME PCR AFTER AUTOMATED DNA ISOLATION**	2013	Belgium	Fecal sample	Microscopy (Ritchie)<br>Multiplex qPCR	Lugol	37	-	-	-	-	-	100	100	10.2143/acb.3170

**Evaluation of the EasyScreen™ Enteric Parasite Detection Kit for the detection of Blastocystis spp., Cryptosporidium spp., Dientamoeba fragilis, Entamoeba complex, and Giardia intestinalis from clinical stool samples**	2013	Australia	Fecal sample	Microscopy<br>-<br>RT-PCR / PCR	hematoxylin modified iron	55	95	-	-	-	Easyscreen™	89 / 100	100 / 100	10.1016/j.diagmicrobio.2013.10.013
**Molecular investigation of zoonotic genotypes of Giardia intestinalis isolates in humans, dogs and cats, sheep, goats and cattle in Araçatuba (São Paulo State, Brazil) by the analysis of ß-giardin gene fragments**	2013	Brazil	Fecal sample	Microscopy<br>(Faust)	-	-	-	-	-	-	-	-	-	10.4081/mr.2013.e6
**IMPACT OF HELICOBACTER PYLORI-GIARDIASIS COINFECTION ON CHILDREN WITH RECURRENT ABDOMINAL PAIN**	2013	Egypt	Fecal sample	Microscopy<br>- 	-	-	-	-	-	-	-	-	-	10.21608/jesp.2013.94832
**Investigation into Cryptosporidium and Giardia in bivalve mollusks farmed in Sardinia region and destined for human consumption**	2013	Italy	Moluscos	Microscopy<br>-<br>PCR	Lugol / Merifluor	-	-	-	-	-	gdh	-	-	10.4081/ijfs.2013.e26

**Concurrent Infections of Giardia duodenalis, Enterocytozoon bieneusi, and Clostridium difficile in Children during a Cryptosporidiosis Outbreak in a Pediatric Hospital in China**	2013	China	Fecal sample	PCR	-	-	-	-	-	-	tpi	-	-	10.1371/journal.pntd.0002437
**A critical assessment of two real-time PCR assays targeting the (SSU) rRNA and gdh genes for the molecular identification of Giardia intestinalis in a clinical laboratory**	2014	United Kingdom	Fecal sample	Microscopy	Lugol	83	87	Techlab Giardia II / Coris Giardia strip rapid membrane test (RMT)	-	-	Giardia-105T / SSU rRNA	61 / 93	98 / 74	10.1136/jclinpath-2014-202224
				(Parasep / Direct)										
				ELISA / Immunochromatographic										
				qPCR / nPCR										
**SEROLOGICAL VERSUS ANTIGEN DETECTION METHODS FOR GIARDIA DUODENALIS DIAGNOSIS**	2014	Egypt	Fecal sample / serum	Microscopy	-	-	-	-	IgG / IgM	87	87	-	-	10.21608/jesp.2014.90161
				ELISA										
**Correlation of Giardia duodenalis assemblages with clinical and epidemiological data in Cuban children**	2014	Cuba	Fecal sample	Microscopy	Lugol	-	-	-	-	-	-	-	-	10.1016/j.meegid.2014.01.007
				(Ritchie)										
**Intraepithelial Giardia Intestinalis**	2014	Mexico	Fecal sample / Duodenal biopsy / duodenal fluid	Microscopy	-	-	-	-	-	-	-	-	-	10.1097/MD.0000000000000277
				-										

**Molecular Identification of Giardia intestinalis in Patients with Dyspepsia**	2014	Egypt	Fecal sample / duodenal fluid	Microscopy<br>(Ritchie / Sheather's sugar flotation)	Methylene blue / Lugol / trichrome	-	-	-	-	-	-	-	-	10.1159/000362644
**Molecular characterisation of Giardia intestinalis assemblages from human isolates at a tertiary care centre of India**	2014	India	Fecal sample	Microscopy<br>(Direct / Ritchie)	Lugol	-	-	-	-	-	-	-	-	10.4103/0255-0857.124290
**Influence of selected stool concentration techniques on the effectiveness of PCR examination in Giardia intestinalis diagnostics**	2014	Poland	Fecal sample	Microscopy<br>Semi-nested PCR<br>Willis	-	-	-	-	-	-	gdh	-	-	10.2478/pjvs-2014-0003
**Detection of Giardia duodenalis assemblage A and B isolates by immunochromatography in stool samples from Rwandan children**	2014	Germany / Rwanda	Fecal sample	Microscopy<br>-<br>Multiplex qPCR	-	29	-	-	-	-	-	50	96	10.1111/1469-0691.12596
**Detection of Cryptosporidium and Giardia in agricultural and water environments in the Qinghai area of China by IFT and PCR**	2014	China	Water sample	Microscopy<br>(Immunofluorescence)<br>Semi-nested PCR	anti-Giardia	-	-	-	-	-	gdh	-	-	10.1007/s00436-014-3979-5
**Molecular Identification of Giardia intestinalis in Patients with Dyspepsia**	2014	Egypt	Fecal sample	PCR	-	-	-	-	-	-	tpi	-	-	10.1159/000362644

**Molecular detection of giardiasis among children at Cairo University Pediatrics Hospitals**	2015	Egypt	Fecal sample	Microscopy (Direct / Ritchie)	Lugol	76	78	RidaQuick	78	96	18s / tpi	-	-	10.1007/s12639-015-0714-9
				Immunochromatographic										
				Nested-PCR / PCR										
**Simultaneous detection of Entamoeba histolytica/dispar, Giardia duodenalis and cryptosporidia by immunochromatographic assay in stool samples from patients living in the Greater Cairo Region, Egypt**	2015	Egypt	Fecal sample	Microscopy (Direct / Paraprep)	Lugol / -	-	-	RidaQuick	-	-	-	-	-	10.1007/s11274-015-1875-5
				Immunochromatographic										
**Comparison of four rapid diagnostic tests, ELISA, microscopy and PCR for the detection of Giardia lamblia, Cryptosporidium spp. and Entamoeba histolytica in feces**	2015	Belgium	Fecal sample	Microscopy (Direct / Ritchie)	iron-hematoxylin	90	100	ProSpecT / ImmunoCard STAT! / Crypto/Giardia Duo-Strip / RidaQuick / Giardia/Cryptosporidium Quik Chek	100 (ELISA) / 58 / 83 / 100	100	-	-	-	10.1016/j.mimet.2015.01.016
				ELISA / Immunochromatographic										
				qPCR										
**Detection and molecular characterisation of Giardia duodenalis, Cryptosporidium spp. and Entamoeba spp. among patients with gastrointestinal symptoms in Gambo Hospital, Oromia Region, southern Ethiopia**	2015	Ethiopia	Fecal sample	Microscopy	-	-	-	Cer Test	-	-	SSU rRNA	-	-	10.1111/tmi.12535

				(Direct)										
				Immunochromatographic										
				qPCR										
**Low sensitivity of the ImmunocardSTAT® Crypto/Giardia Rapid Assay test for the detection of Giardia and Cryptosporidium in fecal samples from children living in Libreville, Central Africa**	2015	Gabon	Fecal sample	Microscopy<br>-	merthiolate-iodine-formaldehyde	-	-	Immunocard STAT	63	96	-	-	-	10.1007/s12639-015-0645-5
				Immunochromatographic										
**Emergent and re-emergent parasites in HIV-infected children: immunological and socio-environmental conditions that are involved in the transmission of Giardia spp. and Cryptosporidium spp.**	2015	Brazil	Fecal sample	Microscopy<br>(Coproplus)	-	-	-	-	-	-	-	-	-	10.1590/0037-8682-0119-2015
				ELISA										
**Investigation of Volatile Organic Compounds Emitted from Faeces for the Diagnosis of Giardiasis.**	2015	United Kingdom	Fecal sample	ELISA / Immunochromatographic	-	-	-	GIARDIA/CRYPTOSPORIDIUM CHEK / RidaQuick	-	-	-	-	-	10.15403/jgld.2014.1121.243.abo
				Gas Chromatography-Mass Spectrometry										
**Dog's genotype of Giardia duodenalis in human: first evidence in Europe**	2015	Slovakia	Fecal sample	Microscopy<br>(Faust)	-	-	-	-	-	-	tpi	-	-	10.1515/ap-2015-0113
				nPCR										

**Molecular diagnosis and genotype analysis of Giardia duodenalis in asymptomatic children from a rural area in central Colombia**	2015	Colombia	Fecal sample	Microscopy (Direct)	-	-	-	-	-	-	SSU rDNA / gdh / tpi	15	95	10.1016/j.meegid.2015.03.015
				Semi-nested PCR / Nested-PCR										
**Giardia Assemblages A and B in Diarrheic Patients: A Comparative Study in Egyptian Children and Adults**	2015	Egypt	Fecal sample	Microscopy (Direct / Ritchie)	-	-	-	-	-	-	bga	-	-	10.1645/14-676
				PCR										
**Comparison of sensitivity and specificity of 4 methods for detection of Giardia duodenalis in feces: immunofluorescence and PCR are superior to microscopy of concentrated iodine-stained samples**	2015	Denmark	Fecal sample	Microscopy (Ritchie / Sheather’s / Immunofluorescence)	Lugol	31 / 67 / 50	100 / 100 / 99	-	-	-	-	91	95	10.1016/j.diagmicrobio.2015.11.005
				qPCR										
**Multiplex PCR for the detection and quantification of zoonotic taxa of Giardia, Cryptosporidium and Toxoplasma in wastewater and mussels**	2015	Turkey / Italy	Water sample / Mussel	qPCR / Multiplex qPCR	-	-	-	-	-	-	bga	-	-	10.1016/j.mcp.2015.01.001

**Frequency, diagnostic performance of coproantigen detection and genotyping of the Giardia among patients referred to a multi-level teaching hospital in northern India**	2016	India	Fecal sample	ELISA	-	-	-	NovaTec	91	91	-	-	-	10.1080/20477724.2016.1254141
**Performance of microscopy and ELISA for diagnosing Giardia duodenalis infection in different pediatric groups**	2016	Brazil	Fecal sample	Microscopy	-	55	100	Wampole II	97	100	bg / gdh	-	-	10.1016/j.parint.2016.08.012
				(Direct / Faust)										
				ELISA										
				nPCR / PCR										
**IMMUNE-MOLECULAR IDENTIFICATION OF GIARDIA INTESTINALIS IN DIARRHOEAL CHILDREN: COMPARISON OF THREE DIAGNOSTIC METHODS.**	2016	Egypt	Fecal sample	Microscopy	Lugol	42 / 73	100 / 100	Ridascreen	-	-	bg	100	91	10.12816/0031632
				(Direct / Ritchie)										
				ELISA										
				nPCR										
**Comparative Evaluation of Three Methods (Microscopic Examination, Direct Fluorescent Antibody Assay, and Immunochromatographic Method) for the Diagnosis of Giardia intestinalis From Stool Specimens**	2016	Turkey	Fecal sample	Microscopy	Lugol / Cellabs, Crypto/Giardia-Cel	100	100	RidaQuick	96	100	-	-	-	10.5152/tpd.2016.4366
				(Ritchie / Immunofluorescence)										
				Immunochromatographic										

**Diagnosis of Giardia duodenalis Infection using Dot Blot in Comparison with Microscopy.**	2016	iran	Fecal sample	Microscopy (Direct / Ritchie)<br>DOT BLOT	-	-	-	Giardia cyst antigen	77 / 97	64 /64	-	-	-	10.2174/1871526516666160714144843
**GIARDIA INTESTINALIS: EVALUATION OF ELISA COPROANTIGEN IN DIAGNOSIS AND EFFECT OF NITAZOXANIDE AND METRONIDAZOLE IN TREATMENT OF GIARDIASIS IN CHILDREN**	2016	Egypt	Fecal sample	Microscopy (Direct / Ritchie)<br>ELISA	Lugol	-	-	Ridascreen	94	85	-	-	-	10.12816/0029122
**Molecular seasonality of Giardia lamblia in a cohort of Egyptian children: a circannual pattern**	2016	Egypt	Fecal sample	Microscopy (Direct / Ritchie)<br>nPCR	-	79	99	-	-	-	bg	-	-	10.1007/s00436-016-5199-7
**Spatial and Molecular Epidemiology of Giardia intestinalis Deep in the Amazon, Brazil**	2016	Brazil	Fecal sample	Microscopy (Ritchie)	-	-	-	-	-	-	-	-	-	10.1371/journal.pone.0158805
**Giardia duodenalis and Giardia enterica in children: first evidence of assemblages A and B in Eastern Slovakia**	2016	Slovakia	Fecal sample	Microscopy (Faust)	-	-	-	-	-	-	-	-	-	10.1007/s00436-016-4935-3
**Molecular Characterization of Cryptosporidium Species and Giardia duodenalis from Symptomatic Cambodian Children**	2016	Cambodia	Fecal sample	Microscopy	-	88	87	-	-	-	bg	-	-	10.1371/journal.pntd.0004822

				(Direct / Evergreen)<br>PCR										
**Evaluation of some micronutrients in patients infected with Giardia lamblia in Hawler city**	2016	Iraq	Fecal sample	Microscopy<br>(Direct)	-	-	-	-	-	-	-	-	-	10.15218/zjms.2016.0013
**Fascinating Giardia Lamblia and Vexing Chronic Spontaneous Urticaria: An Under-appreciated Association**	2016	Pakistan	Fecal sample	Microscopy	-	-	-	-	-	-	-	-	-	10.4172/2155-6121.1000241
**Evaluation of five commercial methods for the extraction and purification of DNA from human faecal samples for downstream molecular detection of the enteric protozoan parasites Cryptosporidium spp., Giardia duodenalis, and Entamoeba spp**	2016	Spain	Fecal sample	qPCR	-	-	-	-	-	-	-	100	100	10.1016/j.mimet.2016.05.020
**Stool antigen immunodetection for diagnosis of Giardia duodenalis infection in human subjects with HIV and cancer**	2017	iran	Fecal sample	Microscopy<br>(Direct)<br>ELISA<br>PCR-RFLP	Lugol / Trichrome	36 / 45	100	Giardia Antigen (Catalog number 7069; Biomerica)	100	100	gdh / tpi	-	-	10.1016/j.mimet.2017.07.004
**Comparison of microscopy, ELISA, and real-time PCR for detectionof Giardia intestinalis in human stool specimens**	2017	Turkey	Fecal sample	Microscopy<br>(Ritchie)<br>ELISA<br>RT-PCR	Lugol / -	50	100	CELISA	100	79	LightMix	-	-	10.3906/sag-1612-71

**Giardia duodenalis infection among rural communities in Yemen: A community-based assessment of the prevalence and associated risk factors**	2017	Yemen	Fecal sample	Microscopy<br>(Direct / Ritchie)	Trichrome	-	-	-	-	-	-	-	-	10.1016/j.apjtm.2017.09.011
**Cytology Preparations of Formalin Fixative Aid Detection of Giardia in Duodenal Biopsy Samples**	2017	United States	duodenal biopsy	Microscopy<br>(Direct)	Pap smear	-	-	-	-	-	-	-	-	10.1097/pas.0000000000000817
**Evaluation of a Real-Time Polymerase Chain Reaction for the Laboratory Diagnosis of Giardia intestinalis in Stool Samples from Schoolchildren from the Centre-Ouest and Plateau Central Regions of Burkina Faso**	2017	Burkina Faso	Fecal sample	Microscopy<br>(Direct / Ritchie)<br>qPCR	-	-	-	-	-	-	SSU rRNA	76	96	10.4172/2471-9315.1000126
**The influence of serial fecal sampling on the diagnosis of giardiasis in humans, dogs, and cats**	2017	Brazil	Fecal sample	Microscopy<br>(Faust)	Lugol	-	-	-	-	-	-	-	-	10.1590/s1678-9946201759061
**Highly sensitive and specific detection of Giardia duodenalis, Entamoeba histolytica, and Cryptosporidium spp. in human stool samples by the BD MAX™ Enteric Parasite Panel**	2017	Germany	Fecal sample	qPCR	-	-	-	-	-	-	SSU rRNA	97	100	10.1007/s00436-017-5720-7

**Multiplex PCR detection of Cryptosporidium sp, Giardia lamblia and Entamoeba histolytica directly from dried stool samples from Guinea-Bissauan children with diarrhoea**	2017	Guinea-Bissauan	Fecal sample	PCR multiplex	-	-	-	-	-	-	18S rRNA	91	95	10.1080/23744235.2017.1320728
**Assessment of the diagnostic performance of four methods for the detection of Giardia duodenalis in fecal samples from human, canine and feline carriers**	2018	Brazil	Fecal sample	Microscopy	Lugol	-	-	Giardia Stool Antigen Detection Microwell	-	-	bg / tpi	-	-	10.1016/j.mimet.2018.01.001
				(Faust)										
				ELISA / Immunochromatography										
				nPCR										
**Comparison of ELISA, nested PCR and sequencing and a novel qPCR for detection of Giardia isolates from Jordan**	2018	Jordan	Fecal sample	ELISA sandwich	-	-	-	Ridascreen	76	68	gdh / bg	89	82	10.1016/j.exppara.2018.01.011
				nPCR / qPCR										
**Rapid diagnostic tests relying on antigen detection from stool as an efficient point of care testing strategy for giardiasis and cryptosporidiosis? Evaluation of a new immunochromatographic duplex assay**	2018	France	Fecal sample	Microscopy	-	-	-	Crypto/Giardia K-SeT®	100	100	-	-	-	10.1016/j.diagmicrobio.2018.07.012
				(Direct / Bailenger's / MIFC)										
				Immunochromatographic										

**Evaluation of the Roche LightMix Gastro parasites multiplex PCR assay detecting Giardia duodenalis, Entamoeba histolytica, cryptosporidia, Dientamoeba fragilis, and Blastocystis hominis**	2018	Germany	Fecal sample	Microscopy	-	-	-	Ridascreen	-	-	LMAGP	-	-	10.1016/j.cmi.2018.03.025
				(Ritchie)										
				ELISA										
				qPCR multiplex										
**Molecular identification of Giardia intestinalis in two cities of the Colombian Caribbean Coast**	2018	Colombia	Fecal sample	Microscopy	Lugol	-	-	-	-	-	-	-	-	10.1016/j.exppara.2018.04.006
				-										
**Spatial distribution of Giardia lamblia infection among general population in Mazandaran Province, north of Iran**	2018	iran	Fecal sample	Microscopy	Lugol / Trichrome	-	-	-	-	-	-	-	-	10.1007/s12639-018-0976-0
				(Ritchie)										
**Effectiveness and Tolerability of 3-Day Mebendazole Treatment of Giardia duodenalis Infection in Adults and Children: Two Prospective, Open-Label Phase IV Trials**	2018	Cuba	Fecal sample	Microscopy	-	-	-	-	-	-	-	-	-	10.1016/j.curtheres.2018.11.002
				(Ritchie)										
**Preliminary Comparison of an in-House Real-Time PCR with the Automated BD Max Enteric Parasite Panel for the Detection of Giardia intestinalis**	2018	Italy	Fecal sample	RT-PCR / BD Max Enteric Parasite Panel (EPP)	-	-	-	-	-	-	SSU rRNA	-	-	10.1645/17-149

**Prevalence and molecular characterization of Strongyloides stercoralis, Giardia duodenalis, Cryptosporidium spp., and Blastocystis spp. isolates in school children in Cubal, Western Angola**	2018	Angola	Fecal sample	qPCR	-	-	-	-	-	-	SSU rRNA	-	-	10.1186/s13071-018-2640-z
**Development of Molecular Diagnosis Using Multiplex Real-Time PCR and T4 Phage Internal Control to Simultaneously Detect Cryptosporidium parvum, Giardia lamblia, and Cyclospora cayetanensis from Human Stool Samples**	2018	Korea	Fecal sample	qPCR / multiplex qPCR	-	-	-	-	-	-	gdh	-	-	10.3347/kjp.2018.56.5.419
**Giardia intestinalis and Fructose Malabsorption: A Frequent Association**	2019	Spain	Fecal sample / Spittle	Immunochromatography / ELISA	-	-	-	Operon / sIgA	-	-	-	-	-	10.3390/nu11122973
**Performance of three rapid diagnostic tests for the detection of Cryptosporidium spp. and Giardia duodenalis in children with severe acute malnutrition and diarrhoea**	2019	Malawi / Kenya	Fecal sample	Immunochromatography	-	-	-	QUIK-CHEK / Rida-Quick / DUO-Strip	70 / 50 / 57	98 / 92 / 95	-	-	-	10.1186/s40249-019-0609-6
**EVALUATION OF NANO-GRAPHENE BASED SANDWICH AND DOT-ELISA AS PROMISING TECHNIQUES FOR DIAGNOSIS OF HUMAN INTESTINAL GIARDIASIS**	2019	Egypt	Fecal sample	Microscopy	Lugol	-	-	CerTest	57 / 83 / 91	77 / 71 / 80	-	-	-	10.21608/jesp.2019.68058

				(Direct / MIFC / Parasep)<br>Sandwich ELISA / Nano graphene based Sandwich ELISA / Dot-ELISA										
**Comparative performance evaluation of four commercial multiplex real-time PCR assays for the detection of the diarrhoea-causing protozoa Cryptosporidium hominis/parvum, Giardia duodenalis and Entamoeba histolytica**	2019	Spain	Fecal sample	multiplex PCR	-	-	-	-	-	-	Gastroenteritis/Parasite Panel I / RIDAGENE / Allplex Gastrointestinal Parasite Panel 4 / FTD Stool Parasites	92 / 95	100 / 99	10.1371/journal.pone.0215068
**Giardia lamblia miRNAs as a new diagnostic tool for human giardiasis**	2019	Israel	duodenal biopsy / Fecal sample	qRT-PCR	-	-	-	-	-	-	miR5 miR6	66	90	10.1371/journal.pntd.0007398
**Identification of volatile biomarkers of Giardia duodenalis infection in children with persistent diarrhoea**	2019	Spain	Fecal sample	Chromatography/mass spectrometry (GC/MS)	-	-	-	-	-	-	-	-	-	10.1007/s00436-019-06433-4
**Specific IgG and IgA Antibody Reactivities in Sera of Children by Enzyme-Linked Immunoassay and Comparison With Giardia duodenalis Diagnosis in Feces**	2020	Brazil	Fecal sample	Microscopy<br>(Lutz / Faust)<br>ELISA	-	-	-	Ridascreen / Anti-Giardia IgG and IgA detection	IgG 80 / IgA 80	IgG 90 / IgA 83	-	-	-	10.3343/alm.2020.40.5.382
**Detection of Giardia lamblia by Microscopic Examination, Rapid Chromatographic Immunoassay Test, and Molecular Technique**	2020	Saudi Arabia	Fecal sample	Microscopy	Lugol / trichrome	52	98	ImmunoCard STAT! / CerTest	59 / 42	89 / 98	SSU rRNA	95	74	10.7759/cureus.10287

				(Direct / Ritchie)<br>Immunochromatography / ELISA<br>qPCR										
**Combined Mini-Parasep SF and Nanogold Immunoassay Show Potential in Stool Antigen Immunodetection for Giardiasis Diagnosis**	2020	Egypt	Fecal sample	Microscopy<br>(Direct / MIFC / Mini-Parasep SF)<br>ELISA	-	-	-	Nanogold Immunoassay	92	94	-	-	-	10.1038/s41598-019-55492-1
**EVALUATION OF POINT OF CARE TEST FOR DIAGNOSIS OF HUMAN GIARDIASIS**	2020	Egypt	Fecal sample	Microscopy<br>(Direct)<br>Immunochromatographic / ELISA	Lugol	75	100	RidaQuick / Ridascreen	95	78	-	-	-	10.21608/asmj.2020.125644
**Uji Validitas dan Efikasi Crypto/Giardia Duo-Strip dalam Mendeteksi Cryptosporidium spp. di Desa Tulehu, Kabupaten Maluku Tengah, Provinsi Maluku**	2020	Indonesia	Fecal sample	Immunochromatographic	-	-	-	Duo-Strip	38	78	-	-	-	10.22146/jkesvo.55361
**Genotyping of Giardia duodenalis in children in upper Egypt using assemblage- specific PCR technique**	2020	Egypt	Fecal sample	Microscopy<br>(Direct)<br>PCR	Lugol	-	-	-	-	-	tpi	-	-	10.1371/journal.pone.0240119
**Examination of Giardia intestinalis with Direct Microscopy and Direct Fluorescent Antibody in Patients with Diarrhea**	2020	Turkey	Fecal sample	Microscopy<br>(Direct / Fluorescence)	Lugol / Meriflour	44	99	-	-	-	-	-	-	10.4274/tpd.galenos.2020.6876

**Comparison of Microscopy and PCR for Detection of Giardia Lamblia and Entamoeba Histolytica in Human Stool Specimens in a Resource Limited Setting in Western Kenya**	2020	Kenya	Fecal sample	Microscopy<br>(Direct / Ritchie)<br>Semi-nested PCR	hematoxylin / Lugol	64	86	-	-	-	gdh	100	100	10.4314/ejhs.v30i6.6
**Infections with Giardia duodenalis and Entamoeba histolytica/Entamoeba dispar as Hidden and Prevalent Conditions in Periurban Communities in the State of Rio de Janeiro, Brazil**	2020	Brazil	Fecal sample	Microscopy<br>(Ritchie)	-	-	-	-	-	-	-	-	-	10.1155/2020/3134849
**Role of Polymerase Chain Reaction in Stool and Duodenal Biopsy for Diagnosis of Giardiasis in Patients with Persistent/Chronic Diarrhea**	2020	India	Fecal sample / duodenal biopsy	Microscopy<br>(Direct)<br>nPCR	Giemsa	93	100	-	-	-	nPCR	100	94	10.1007/s10620-019-06042-2
**Molecular detection of Tropheryma whipplei, Cryptosporidium spp., and Giardia lamblia among celiac disease samples**	2020	iran	duodenal biopsy	Microscopy<br>-<br>Multiplex PCR	Hematoxylin and eosin	-	-	-	-	-	tpi	-	-	10.4103/jrms.jrms_487_19
**Detection of Cryptosporidium and Giardia in foods of plant origin in North-Western Greece**	2020	Greece	Vegetables and fruits	Microscopy<br>(Ritchie)	Trichrome	-	-	-	-	-	-	-	-	10.2166/wh.2020.037

**Rectal Swabs as an Alternative Sample Collection Method to Bulk Stool for the Real-Time PCR Detection of Giardia duodenalis**	2020	Niger	rectal swab	Multi-parallel qPCR	-	-	-	-	-	-	ribosomal RNA gene	78	-	10.4269/ajtmh.19-0909
**Giardiosis en población pediátrica de la provincia de Castellón: clínica e impacto**	2020	Spain	-	qPCR	-	-	-	-	-	-	SSU rRNA	-	-	10.1016/j.anpedi.2020.06.023
**A large outbreak of giardiasis in a municipality of the Bologna province, north-eastern Italy, November 2018 to April 2019**	2021	Italy	Fecal sample	Microscopy	Lugol	-	-	ImmunoCard STAT!	-	-	-	-	-	10.2807/1560-7917.es.2021.26.35.2001331
				-										
				Immunochromatographic										
**Intestinal giardiasis in children: Five years' experience in a reference unit**	2021	Spain	Fecal sample	Microscopy (Direct / Parasep®)	-	-	-	Operon	-	-	-	-	-	10.1016/j.tmaid.2021.102082
				Immunochromatographic										
**Sparse Evidence for Giardia intestinalis, Cryptosporidium spp. and Microsporidia Infections in Humans, Domesticated Animals and Wild Nonhuman Primates Sharing a Farm–Forest Mosaic Landscape in Western Uganda**	2021	Uganda	Fecal sample	Immunochromatographic	-	-	-	RidaQuick	-	-	tpi	-	-	10.3390/pathogens10080933
				nPCR										
**FEASIBILITY OF A RAPID LATERAL FLOW TEST FOR SIMULTANEOUS DETECTION OF GIARDIA LAMBLIA AND**	2021	Egypt	Fecal sample / duodenal fluid	Microscopy	-	80 / 30	100 / 100	RidaQuick	96	99	-	-	-	10.21608/jesp.2021.193310

**CRYPTOSPORIDIUM PARVUM IN DUODENAL ASPIRATES OF PATIENTS SUFFERING FROM CHRONIC LIVER DISEASES AND ELIGIBLE FOR UPPER ENDOSCOPY**				(Direct / Ritchie / Fluorescence)<br>Immunochromatographic										
**Efficacy of triage parasite panel in diagnosis of Entamoeba histolytica, Giardia lamblia, and Cryptosporidium parvum antigens in symptomatic children stool specimens**	2021	Egypt	Fecal sample	Microscopy<br>(Direct / Ritchie)<br>Immunochromatographic / ELISA	Lugol / -	76	88	RidaQuick / TechLab Giardia II (PT5012)	76 / 70	84 / 88	-	-	-	10.21608/evmspj.2021.192801
**Comparative analysis of routine parasitological methods for recovery of cysts, molecular detection, and genotyping of Giardia duodenalis**	2021	Brazil	Fecal sample	Microscopy<br>(Lutz / Faust / Ritchie)<br>Semi-nested PCR	-	-	-	-	-	-	gdh	-	-	10.1007/s10096-021-04280-9
**Molecular detection of glutamate dehydrogenase gene of Giardia lamblia isolated from food handlers in Erbil city**	2021	Iraq	Fecal sample	Microscopy<br>(Direct)<br>nPCR	-	-	-	-	-	-	gdh	-	-	10.15218/zjms.2021.004

**Optimization and validation of a loop-mediated isothermal amplification (LAMP) assay for detection of Giardia duodenalis in leafy greens**	2021	Nepal / Dominican Republic	Green leaves	LAMP / nPCR	-	-	-	-	-	-	EF1α / SSU rRNA	-	-	10.1016/j.fawpar.2021.e00123
**Commercial Simplex and Multiplex PCR Assays for the Detection of Intestinal Parasites Giardia intestinalis, Entamoeba spp., and Cryptosporidium spp.: Comparative Evaluation of Seven Commercial PCR Kits with Routine In-House Simplex PCR Assays**	2021	France	Fecal sample	SimpPCRa / MultPCRa	-	-	-	-	-	-	CerTest / FAST-TRACK FTD / DIAGENODE	96 / 90 / 76	93 / 92 / 97	10.3390/microorganisms9112325
**Giardiasis in a paediatric population of the province of Castellon. Clinical details and impact**	2021	Spain	Fecal sample	qPCR	-	-	-	-	-	-	SSU rRNA	-	-	10.1016/j.anpede.2020.06.010
**Diagnosis of Blastocystis sp., Cryptosporidium sp., and Giardia intestinalis by Multiplex PCR: An Optimization Study**	2021	Turkey	Fecal sample	PCR / Multiplex PCR	-	-	-	-	-	-	tpi	-	-	10.5222/tmcd.2021.20981
**Giardia duodenalis: Detection by Quantitative Real-Time PCR and Molecular Diversity**	2021	Spain	Fecal sample	qPCR	-	-	-	-	-	-	gdh / bg / tpi	-	-	10.1007/978-1-0716-1681-9_6
**Possible Correlation between Giardia duodenalis Genotypes and Fecal Calprotectin in Children with Diarrhea**	2022	Egypt	Fecal sample	ELISA	-	-	-	PhiCal Calprotectin	100	100	bg	80	100	10.18502/ijpa.v17i4.11275
				nPCR										
**Role of anti-Giardia recombinant cyst wall protein IgG polyclonal antibodies in**	2022	Egypt	Fecal sample	sandwich ELISA	-	-	-	Anti-G. duodenalis IgG pAbs	97	92	-	-	-	10.1186/s13568-022-01484-w

**diagnosis and protection**														
**Incidence, Management Experience and Characteristics of Patients with Giardiasis and Common Variable Immunodeficiency**	2022	Spain	Fecal sample	Microscopy	-	-	-	Rida Quick	-	-	-	-	-	10.3390/jcm11237007
				-										
				Immunochromatographic										
				Multiplex PCR										
**Intestinal Giardiasis in Children Undergoing Upper Endoscopy for Unexplained Gastrointestinal Symptoms: Implication for Diagnosis**	2022	Egypt	Fecal sample	Microscopy	-	-	-	-	90	-	-	-	-	10.1080/15513815.2022.2053013
				-										
				sandwich ELISA										
**Investigation of the Precence of <i>Entamoeba histolytica, Giardia intestinalis</i> and <i>Cryptosporidium</i> spp. in Patients Who Undergone Endoscopy and Colonoscopy**	2022	Turkey	Fecal sample / Duodenal swab	Microscopy	Lugol	-	-	DRG® ELISA	90	-	-	-	-	10.4274/tpd.galenos.2022.30502
				(Direct)										
				ELISA										
**Evaluación de pruebas inmunológicas en el diagnóstico de Giardia duodenalis y Cryptosporidium spp., Honduras**	2022	Honduras	Fecal sample	Microscopy	Lugol / Trichrome	57 / 73	100 / 100	IVD / ImmunoCard STAT!	100 / 83	83 / 96	-	-	-	10.5377/rmh.v90i1.14394
				(Direct)										
				EISA / Immunochromatographic										

**Evaluation of IL-17 and IL-35 in patients with giardiasis in Thi-Qar province, Iraq**	2022	Iraq	Fecal sample	Microscopy<br>(Direct)	Lugol	-	-	-	-	-	-	-	-	10.25122/jml-2021-0328
**Extraction of the DNA of Giardia lamblia isolated from vegetables and fruits in a simplified way and its diagnosis using Nested-PCR**	2022	Iraq	Vegetables and fruits	Microscopy<br>(Direct)<br>nPCR	Lugol	-	-	-	-	-	-	-	-	10.1007/s12639-022-01484-4
**Occurrence of Cryptosporidium spp. and Giardia spp. Infection in Humans in Latvia: Evidence of Underdiagnosed and Underreported Cases**	2022	Latvia	Fecal sample	Microscopy<br>(Willis / immunofluorescence)	AquaGlo	-	-	-	-	-	-	-	-	10.3390/medicina58040471
**Giardiasis and cryptosporidiosis in children and dogs, and the first report of assemblage E in northeastern Brazil**	2022	Brazil	Fecal sample	Microscopy<br>(TF-TestTM / Faust)<br>nPCR	Lugol	-	-	-	-	-	SSU rRNA / bg / gdh / tpi	-	-	10.21203/rs.3.rs-1731287/v1
**Evaluation of a Novel Commercial Real-Time PCR Assay for the Simultaneous Detection of Cryptosporidium spp., Giardia duodenalis, and Entamoeba histolytica**	2022	Spain	Fecal sample	qPCR	-	-	-	-	-	-	18S rRNA	94	100	10.1128/spectrum.00531-22
**Comparative Evaluation of Real-Time Screening PCR Assays for Giardia duodenalis and of Assays**	2022	ghana	Fecal sample	qPCR	-	-	-	-	-	-	18S rRNA / gdh / bg	100 / 17 / 31	100 / 92 / 100	10.3390/microorganisms10071310

**Discriminating the Assemblages A and B**														
**Development, Optimisation and Validation of a Novel Multiplex Real-Time PCR Method for the Simultaneous Detection of Cryptosporidium spp., Giardia duodenalis and Dientamoeba fragilis**	2022	Spain	Fecal sample	qPCR Multiplex	-	-	-	-	-	-	SSU rRNA	97	100	10.3390/pathogens11111277
**Molecular detection of Giardia intestinalis in fresh vegetables and watercourses of Giza, Egypt**	2022	Egypt	Vegetable	PCR	-	-	-	-	-	-	18S rRNA	-	-	10.21608/ejabf.2022.239742
**Giardiasis in children and dogs, and the first report of assemblage E in dogs from northeastern Brazil**	2023	Brazil	Fecal sample	Microscopy<br>(Hoffmann / Faust)<br>nPCR	Lugol	-	-	-	-	-	SSU rRNA / bg / gdh / tpi	-	-	10.1590/s1984-29612023010
**Giardia lamblia-infected preschoolers present growth delays independent of the assemblage A, B or E**	2023	Brazil	Fecal sample	Microscopy<br>(Ritchie)<br>PCR	-	-	-	-	-	-	gdh / bg	-	-	10.1590/0074-02760230043

Supplementary Table S2 – Quantification of types of diagnostic techniques employed in each country.

Country	Immune	Molecular	Microscopy
Australia	1	3	2
Angola	0	1	0
Bangladesh	1	0	1
Belgium	1	2	2
Brazil	6	6	18
Burkina Faso	0	1	1
Canada	0	1	3
Cambodia	0	1	1
China	0	2	1
Colombia	2	1	4
Costa do Marfim	0	0	1
Cuba	0	0	3
Dominican Republic	0	1	0
Denmark	0	1	1
Egypt	16	9	20
Ethiopia	0	0	1
France	1	2	2
Gabon	1	0	1
ghana	0	1	0
Germany	2	4	4
Greece	0	0	1
Guinea-Bissauan	0	1	0
Honduras	1	0	1
India	1	2	3
Iran	2	2	4
Iraq	0	2	4
Israel	0	1	0
Italy	3	4	3
Japan	0	0	1
Jordan	0	1	1
Kenya	1	1	1
Korea	0	1	0
Latvia	0	0	1

Malawi	1	0	0
Mexico	0	0	1
Nepal	0	1	1
Netherlands	1	1	1
Niger	0	1	0
Nigeria	0	1	0
Norway	2	0	4
Poland	3	1	3
Portugal	1	0	1
Rwanda	0	1	1
Saudi Arabia	1	0	1
Slovakia	0	1	3
Spain	6	11	4
Sweden	1	0	1
Thailand	2	2	3
Turkey	3	4	5
Uganda	1	1	0
United Kingdom	2	1	1
United States	0	0	1
Venezuela	1	0	1
Yemen	0	0	1
